# Exploring the diversity of pyruvate:ferredoxin oxidoreductases

**DOI:** 10.1002/pro.70853

**Published:** 2026-11-14

**Authors:** Colette Maignet‐Magard, Myriam Brugna, Ariane Atteia, Arlette Kpebe, Marianne Guiral, Carole Baffert

**Affiliations:** ^1^ Aix Marseille Univ, CNRS, BIP, IMM, IM2B Marseille France; ^2^ Aix Marseille Univ, CNRS, Centrale Med, iSm2, IM2B Marseille France; ^3^ MARBEC, Univ Montpellier, CNRS, Ifremer, IRD Sète France

**Keywords:** anaerobic metabolism, pyruvate decarboxylation, pyruvate synthase, pyruvate:ferredoxin oxidoreductase, structural diversity, thiamine pyrophosphate

## Abstract

Pyruvate:ferredoxin oxidoreductases (PFORs) are key enzymes in the anaerobic metabolism of numerous diverse organisms. Their significance extends far beyond the oxidative decarboxylation of pyruvate, their canonical reaction. PFORs support diverse physiological roles, including fermentation and carbon assimilation, and have been integrated into a wide range of metabolic networks, linking carbon flux to redox balance in many bacteria and archaea, as well as in selected eukaryotes. PFORs catalyze the reversible conversion of pyruvate to acetyl‐coenzyme A coupled to the reduction of low‐potential ferredoxin. This process relies on a catalytic mechanism involving a thiamine pyrophosphate cofactor and iron–sulfur clusters. The PFOR enzymes exhibit remarkable diversity in terms of structure (subunit composition, domain architecture, and quaternary organization), function and kinetics. This diversity reflects evolutionary adaptations to different biological contexts. In this review, we examine PFOR diversity from molecular and physiological perspectives, with a focus on the enzymes that have been biochemically characterized or at least demonstrated to exhibit PFOR activity.

## 1 INTRODUCTION

Pyruvate:ferredoxin oxidoreductase (PFOR, EC 1.2.7.1) is a representative member of the 2‐oxoacid:ferredoxin oxidoreductase (OFOR) family, a phylogenetically and functionally diverse group of enzymes that also includes 2‐oxoglutarate oxidoreductase (OGOR), 2‐ketoisovalerate oxidoreductase (VOR), indolepyruvate oxidoreductase (IOR) and oxalate oxidoreductase (OOR) (Chen et al., 2018; Cossu et al., 2024; DeFeo et al., 2026; Gibson et al., 2016; Pierce et al., 2017). Since its original description in 1971 in a series of four seminal papers by Rabinowitz and colleagues (Raeburn & Rabinowitz, 1971a, 1971b; Uyeda & Rabinowitz, 1971a, 1971b), PFOR has emerged as a paradigmatic enzyme of anaerobic metabolism. It catalyzes the reversible oxidative decarboxylation of pyruvate to acetyl‐coenzyme A (acetyl‐CoA) and CO_2_, and the release of two low‐potential electrons. These electrons are captured by small‐size redox proteins, most commonly a ferredoxin (Fd). The catalyzed reaction is as follows (Ragsdale, 2003):

pyruvate + CoA + 2Fd_oxidized_ ⇄ CO_2_ + acetyl‐CoA + 2Fd_reduced_.

PFOR relies on a conserved catalytic core that accommodates thiamine pyrophosphate (TPP) and a catalytically essential Mg^2+^ ion, while [4Fe‐4S] (FeS) cluster(s) are involved in internal electron transfer and relay to/from external redox partners. Most PFORs described to date are highly sensitive to dioxygen (O_2_) and are irreversibly inactivated upon exposure to air. Some PFORs derived from hyperthermophilic organisms have been shown to be bifunctional. In addition to the typical PFOR reaction, they catalyze the non‐oxidative decarboxylation of pyruvate in a CoA‐ and TPP‐dependent manner (PDC activity), producing acetaldehyde. This PDC activity does not require Fd in vitro, and a low ratio of oxidized to reduced Fd is predicted to favor acetaldehyde production in vivo (Alharbi et al., 2022; Eram et al., 2014, 2015; Eram & Ma, 2013; Ma et al., 1997; Nisa et al., 2020).

PFORs are broadly distributed across Bacteria and Archaea and found in some Eukaryotes. These enzymes support a variety of metabolic strategies, from fermentative energy conservation to carbon assimilation and redox balancing. Beyond their fundamental biological importance, PFORs have attracted growing interest as potential therapeutic targets against pathogenic bacteria and parasites (Gilles & Hoffman, 2002; Warren et al., 2012). Only a few comprehensive reviews have focused on this enzyme family (Charon et al., 1999a; Gibson et al., 2016; Kerscher & Oesterhelt, 1982; Ragsdale, 2003). Here, we review the diversity of PFORs at the molecular and physiological levels, emphasizing subunit composition, taxonomic distribution, redox partners and functional roles. We also discuss catalytic and structural features considering recent advances. Together, these characteristics underscore the structural and functional plasticity of this enzyme family, framing the comparative analyses developed in the following sections. We focused on enzymes that have been characterized through experimentation or demonstrated to have PFOR activity (Tables 1 and S1).

**TABLE 1:** Enzymes exhibiting PFOR activity.

Organism	Domain of life	Subunits	Quat. state	Locus tag	2‐Oxoacid substrates	Accession NCBI (Uniprot)	References
*Aeropyrum pernix* K1	A	α𝛽	M	APE_2126	Pyruvate 2‐Oxobutyrate (100%) Glyoxylate (89%) 3‐Hydroxypyruvate (38%) 2‐Oxo‐4‐methylthiobutyrate (37%) 2‐Oxoadipate (37%) Phenylpyruvate (8%) 2‐Oxoglutarate (6%)	BAA81137 (Q9YA13)	Nishizawa et al. (2005)
*Aeropyrum pernix* K1	A	α𝛽	M	APE_1473	Pyruvate Glyoxylate (105%) 2‐Oxobutyrate (100%) 2‐Oxoglutarate (99%) 3‐Hydroxypyruvate (57%) 2‐Oxoadipate (52%) 2‐Oxo‐4‐methylthiobutyrate (50%) Phenylpyruvate (38%) 2‐Oxohexanoate (25%) 4‐Hydroxyphenylpyruvate (17%)	BAA80471 (Q9YBX7)	Nishizawa et al. (2005)
*Halobacterium salinarum* (formerly *Halobacterium halobium*) NRC‐1	A	α𝛽	D	VNG_1128G	Pyruvate 2‐Oxobutyrate (140%–154%) 2‐Oxoglutarate (59%–124%)	AAG19514 (Q9HQJ9)	Kerscher and Oesterhelt (1982) and Kerscher and Oesterhelt (1981b)
*Haloferax volcanii* H26	A	α𝛽	D	HVO_1305	Pyruvate	ADE02628 (D4GXF1)	Kuprat et al. (2021)
*Saccharolobus solfataricus* (formerly *Sulfolobus solfataricus*)	A	α𝛽		SSO2815	Pyruvate	AAK42926 (Q97V21)^a^	Alharbi et al. (2022)
*Sulfolobus acidocaldarius* DSM 639	A	α𝛽		Saci_2306	Pyruvate 2‐Oxoglutarate (20%)	AAY81592 (Q4J6I9)	Alharbi et al. (2022) and Witt et al. (2019)
*Sulfurisphaera tokodaii* (formerly *Sulfolobus tokodaii*) Strain 7	A	OFOR1 α𝛽	D	STK_23000	Pyruvate 2‐Oxobutyrate (95%/118%) 2‐Oxoglutarate (36%/177%)	BAB67411 (Q96Y66)	Fukuda and Wakagi (2002), Iwasaki et al. (1995). Yan et al. (2016), and Zhang et al. (1996)
*Sulfurisphaera tokodaii* (formerly *Sulfolobus tokodaii*) Strain 7	A	OFOR2 α𝛽	D	STK_24350	Pyruvate 2‐Oxoglutarate (20%)	BAB67545 (Q96XT2)	Yan et al. (2016)
*Archaeoglobus fulgidus* DSM 4304	A	α𝛽𝛾𝛿	M	AF_1701	Pyruvate 2‐Oxaloacetate (72%) 2‐Oxobutyrate (30%)	AAB89548.1 (O28572)	Kunow et al. (1995)
*Ignicoccus hospitalis* KIN4/I	A	α𝛽𝛾𝛿		Igni_1258	Pyruvate	ABU82434 (A8ABY2)	Xiao et al. (2021)
*Ignicoccus islandicus* DSM 13165	A	α𝛽𝛾𝛿		EYM_04020	Pyruvate	ALU11715 (A0A0U3EB88)	Xiao et al. (2021)
*Methanococcus maripaludis* JJ/DSM 2067	A	α𝛽𝛾𝛿^b^	D	MMJJ_13330	Pyruvate 2‐Oxaloacetate (88%) 2‐Oxobutyrate (49%) Indol‐3‐pyruvate (4%)	AVB76712.1 (Q9P9E5)	Lin and Whitman (2004) and Lin et al. (2003)
*Methanopyrus kandleri* AV19	A	α𝛽𝛾𝛿		MK0082	Pyruvate	AAM01299 (Q8TZ57)	Xiao et al. (2021)
*Methanosarcina acetivorans* C2A	A	α𝛽𝛾𝛿	D	MA_0032	Pyruvate	AAM03486 (Q8TUN4)	Bonitatibus et al. (2025) and Cossu et al. (2024)
*Methanosarcina barkeri* Fusaro DSM 804	A	α𝛽𝛾𝛿	M	Mbar_A1000	Pyruvate 2‐Oxobutyrate (54%)	AAZ69968 (P80521)	Bock et al. (1997) and Bock et al. (1996)
*Methanothermobacter marburgensis* Str. Marburg (formerly *Methanobacterium thermoautotrophicum*)	A	α𝛽𝛾𝛿		MTBMA_c03140	Pyruvate 2‐Oxobutyrate (40%) 3‐Hydroxypyruvate (7%) Glyoxylate (3%)	ADL57920.1 (P80900)	Tersteegen et al. (1997)
*Pyrococcus furiosus* DSM 3638	A	α𝛽𝛾𝛿		PF0966	Pyruvate 2‐Oxaloacetate (61%) 2‐Oxobutyrate (52%)	AAL81090 (Q51804)	Blamey and Adams (1993), Eram et al. (2014), Ma et al. (1997), Mai and Adams (1994), and Menon et al. (1998)
*Pyrodictium delaneyi* Su06	A	α𝛽𝛾𝛿		Pyrde_0268	Pyruvate	ALL00318 (A0A0P0N2R4)	Xiao et al. (2021)
*Pyrolobus fumarii* DSM 11204	A	α𝛽𝛾𝛿		Pyrfu_0709	Pyruvate	AEM38578 (G0ED19)	Xiao et al. (2021)
*Thermococcus guaymasensis* DSM 11113	A	α𝛽𝛾𝛿	D	‐	Pyruvate	AGM16434 (W8CQR1)	Eram et al. (2014)
*Thermococcus kodakarensis* KOD1	A	α𝛽𝛾𝛿	D	TK1983	Pyruvate	BAD86172 (Q5JIJ7)	Nisa et al. (2020)
*Thermoproteus tenax* DSM 2078	A	α𝛽𝛾𝛿		TTX_0209^c^	Pyruvate	CCC80888 (G4RMU4)	Xiao et al. (2021)
*Anabaena cylindrica* PCC 7122	B	α		Anacy_0789	Pyruvate 2‐Oxobutyrate (11%) 2‐Oxoisovalerate (3%)	AFZ56372 (K9ZCQ2)	Bothe et al. (1974) and Neuer and Bothe (1982)
*Nostoc* sp. PCC 7120 (formerly *Anabaena* sp. PCC 7120)	B	α		–^d^	Pyruvate	–^d^	Razquin et al. (1995)
*Campylobacter jejuni* NCTC 11168	B	α		Cj1476c	Pyruvate	CAL35583 (Q0P8E1)	Kendall et al. (2014) and St. Maurice et al. (2007)
*Citrobacter* sp. S77	B	α	D	CIT_000011	Pyruvate	GAN52676.1 (−)	Takenaka et al. (2017)
*Chlorobaculum tepidum* TLS	B	α	D	CT1628	Pyruvate	AAM72853 (Q8KC02)	Bonitatibus et al. (2025), Bonitatibus et al. (2024), Yoon et al. (2001) and Yoon et al. (1999)
*Clostridium acetobutylicum* DSM 1731	B	α		–^d^	Pyruvate 2‐Oxobutyrate (12%)	–^d^	Meinecke et al. (1989)
*Clostridium sporogenes* NCIB 10696	B	α^e^		–^d^	Pyruvate	–^d^	Payne et al. (1990)
*Gottschalkia acidurici* (formerly *Clostridium acidurici*)	B	α	D	–^d^	Pyruvate	–^d^	Kerscher and Oesterhelt (1982), Raeburn and Rabinowitz (1971a, 1971b), Uyeda and Rabinowitz (1971a, 1971b)
*Desulfocurvibacter africanus* (formerly *Desulfovibrio africanus*) DSM 2603	B	α′	D	Desaf_2186^f^	Pyruvate	EGJ50514 (F3YWM1)^f^	Chabrière et al. (1999), Pieulle et al. (1999), Pieulle et al. (1995), and Witt et al. (2019)
*Desulfomicrobium* sp. Rod‐9	B	α′^e^		–^d^	Pyruvate	–^d^	Kushkevych (2015)
*Desulfovibrio piger* Vib‐7	B	α^e^		–^d^	Pyruvate	–^d^	Kushkevych (2015)
*Nitratidesulfovibrio vulgaris* (formerly *Desulfovibrio vulgaris*) Hildenborough ATCC 29579	B	α′	O	DVU_3025	Pyruvate	AAS97496 (Q726T1)	Garczarek et al. (2007)
*Nitratidesulfovibrio vulgaris* (formerly *Desulfovibrio vulgaris*) Miyazaki F IAM 12604	B	α′^e^		–^d^	Pyruvate 2‐Oxobutyrate (16%)	–^d^	Ogata and Yagi (1986)
*Escherichia coli* K12 BW25113	B	α	D	BW25113_1378	Pyruvate 2‐Oxobutyrate (9%)	AIN31831 (−)	Blaschkowski et al. (1982) and Nakayama et al. (2013)
*Heliomicrobium modesticaldum* Ice1	B	α		HM1_0807	Pyruvate	ABZ83749 (B0TB22)	Walters et al. (2024)
*Klebsiella pneumoniae* M5a1	B	α		–^d^	Pyruvate	–^d^	Wahl and Orme‐Johnson (1987)
*Neomoorella thermoacetica* (formerly *Moorella thermoacetica* or *Clostridium thermoaceticum*) ATCC 39073	B	α	D	Moth_0064	Pyruvate	ABC18403 (Q2RMD6)	Chen et al. (2018), Furdui and Ragsdale (2000), Menon and Ragsdale (1997), and Wahl and Orme‐Johnson (1987)
*Rhodobacter capsulatus* SB 1003	B	α	D	RCAP_rcc01728	Pyruvate	ADE85473 (D5AU34)	Yakunin and Hallenbeck (1998)
*Rhodospirillum rubrum* ATCC 11170	B	α	D	Rru_A2398	Pyruvate	ABC23198 (Q53046)	Brostedt and Nordlund (1991), Edgren and Nordlund (2006), and Leroy et al. (2015)
*Synechocystis* sp. PCC 6803	B	α		sll0741	Pyruvate	BAA10774 (P52965)	Wang et al. (2022)
*Thermoanaerobacter kivui* DSM 2030	B	α (PFOR‐1)	D	TKV_c04340	Pyruvate	AIS51636 (A0A097APA2)	Katsyv, Schoelmerich, et al. (2021)
*Thermoanaerobacterium saccharolyticum* JW/SL‐YS485	B	α		Tsac_0046	Pyruvate	AFK85084 (I3VRD9)	Zhou et al. (2015)
*Magnetococcus marinus* MC‐1	B	α′𝛽′		Mmc1_1741	Pyruvate	ABK44249 (A0L8F6)	Bonitatibus et al. (2025)
*Acetivibrio thermocellus* (formerly *Clostridium thermocellum*) DSM 1313	B	α𝛽𝛾𝛿 (PFOR1) α (PFOR3) α𝛽𝛾𝛿 (PFOR4)^g^		clo1313_0022 (PFOR1) clo1313_0673 (PFOR3) clo1313_1354 (PFOR4)	Pyruvate	ADU73122 (−) (PFOR1) ADU73753 (−) (PFOR3) ADU74417 (−) (PFOR4)	Cui et al. (2019) and Xiong et al. (2016)
*Helicobacter pylori* 26,695	B	α𝛽𝛾𝛿	D	HP_1110	Pyruvate	AAD08154 (O25738)	Hughes et al. (1998), Hughes et al. (1995), and St. Maurice et al. (2007)
*Pseudothermotoga hypogea* (formerly *Thermotoga hypogea*) DSM 11164	B	α𝛽𝛾𝛿		AJ81_03020	Pyruvate	AJC73351 (A0A0X1KQ45)	Eram et al. (2015)
*Thermotoga maritima* DSM 3109	B	α𝛽𝛾𝛿		TM_0017	Pyruvate	AAD35111 (O05651)	Blamey and Adams (1994), Eram et al. (2015), and Kletzin and Adams (1996)
*Aquifex aeolicus* VF5	B	α𝛽𝛾𝛿ε	D	aq_1195	Pyruvate	AAC07211 (O67254)	Prioretti et al. (2023)
*Hydrogenobacter thermophilus* TK‐6	B	α𝛽𝛾𝛿ε	D	HTH_1553	Pyruvate 2‐Oxobutyrate (3%–8%) 2‐Oxaloacetate (3%–8%)	BAI70002 (D3DJK0)	Ikeda et al. (2010), Ikeda et al., 2006, and Yoon et al. (1997)
*Chlamydomonas reinhardtii* CC124	E	α	D	CHLRE_02g095137v5	Pyruvate 2‐Oxaloacetate (80%)	CCA61743 (L8B958)	Noth et al. (2013) and Van Lis et al. (2013)
*Acanthamoeba castellanii* ATCC 30011	E	α		ACA1_210530^h^	Pyruvate	ELR14981 (L8GS75)	Carvalho‐Kelly et al. (2025)
*Entamoeba histolytica* HM‐1:IMSS	E	α		EHI_051060	Pyruvate 2‐Oxobutyrate (89%) 2‐Oxaloacetate (67%)	XP_657019 (C4LTX6)	Pineda et al. (2010) and Reeves et al. (1977)
*Giardia duodenalis* (formerly *Giardia intestinalis*) BRIS/83/HEPU/106	E	α	D	–^d^	Pyruvate 2‐Oxaloacetate (40%) 2‐Oxobutyrate (5%)	–^d^	Townson et al. (1996)
*Trichomonas vaginalis* Bushby/CNCD 188	E	α	D	–	Pyruvate 2‐Oxobutyrate (45%) 2‐Oxoglutarate (5%)	AY661465 (Q4KY23)	Meza‐Cervantez et al. (2011), Steinbüchel and Müller (1986) and Williams et al. (1987)
*Tritrichomonas fœtus* ATCC 30003/ATCC 30924	E	α		–^d^	Pyruvate	–^d^	Docampo et al. (1987) and Steinbüchel and Müller (1986)

*Note*: Domain of life: A stands for Archaea, B for Bacteria, E for Eukaryotes. Subunits: Subunits that constitute the enzyme (see text and Figure 3 for details). Quat. State: Demonstrated quaternary state; M: Monomer, D: Dimer, O: Octomer. Locus tag: Gene encoding subunit α of PFOR. 2‐Oxoacid substrates: Compounds used as substrates with the percentage of activity relative to that obtained with pyruvate shown in parentheses. Accession: Numbers (subunit α) from NCBI and UniProtKB (in parentheses) databases.

^a^ The accession number and the locus tag correspond to strain P2. In Alharbi et al. (2022), the strain studied is not specified.

^b^ It is believed that this PFOR is a four‐subunit enzyme (see Section 3 for details) contrary to what has been previously proposed (Lin et al., 2003).

^c^ The genes from locus tags TTX_0209 and TTX_0210 which code for the α and 𝛽 subunits, and TTX_2035 and TTX_2036, from a second OFOR gene clusters, and which code for the 𝛾 and 𝛿 subunits, have been cloned for PFOR production (Xiao et al., 2021).

^d^ The identity of the PFOR that has been studied or characterized remains unknown due to the presence of multiple PFOR/OFOR genes within the genome.

^e^ The one‐subunit structure (α′ or α) is assumed, as it was not determined when the enzyme was characterized.

^f^ Locus tag and accession numbers are for the strain Walvis Bay.

^g^ A fourth gene cluster (clo1313_0382–0385) encoding an uncharacterized four‐subunit PFOR (PFOR2) is present in the genome.

^h^ Partial sequence.

## 2 STRUCTURE AND CATALYTIC MECHANISM

Structural studies of various PFORs in the presence of substrates have provided molecular insights into the mechanism of OFORs. 3D‐structures are available for six PFORs, those from the bacteria *Desulfocurvibacter africanus* (Cavazza et al., 2006; Chabrière et al., 1999), *Neomoorella thermoacetica* (Chen et al., 2018), *Nitratidesulfovibrio vulgaris* Hildenborough (*Nv*H) (Garczarek et al., 2007), and the archaea *Methanosarcina acetivorans* (Cossu et al., 2024) and *Sulfurisphaera tokodaii* (Yan et al., 2016). The different cofactors are oriented similarly, with the Mg^2+^ ion near the pyrophosphate group of TPP (Figure 1). All structurally characterized PFORs contain three FeS clusters, with the exception of the *S. tokodaii* enzyme (Yan et al., 2016) that contains only the cluster proximal to the TPP. These structural studies have confirmed that a common fold is largely conserved among the various orthologues of the PFOR family despite their differences in domain arrangements and oligomeric states (see Section 3), and regardless of the catalyzed physiological reaction (i.e., the oxidative decarboxylation of pyruvate or the reduction of CO_2_).

**FIGURE 1:**
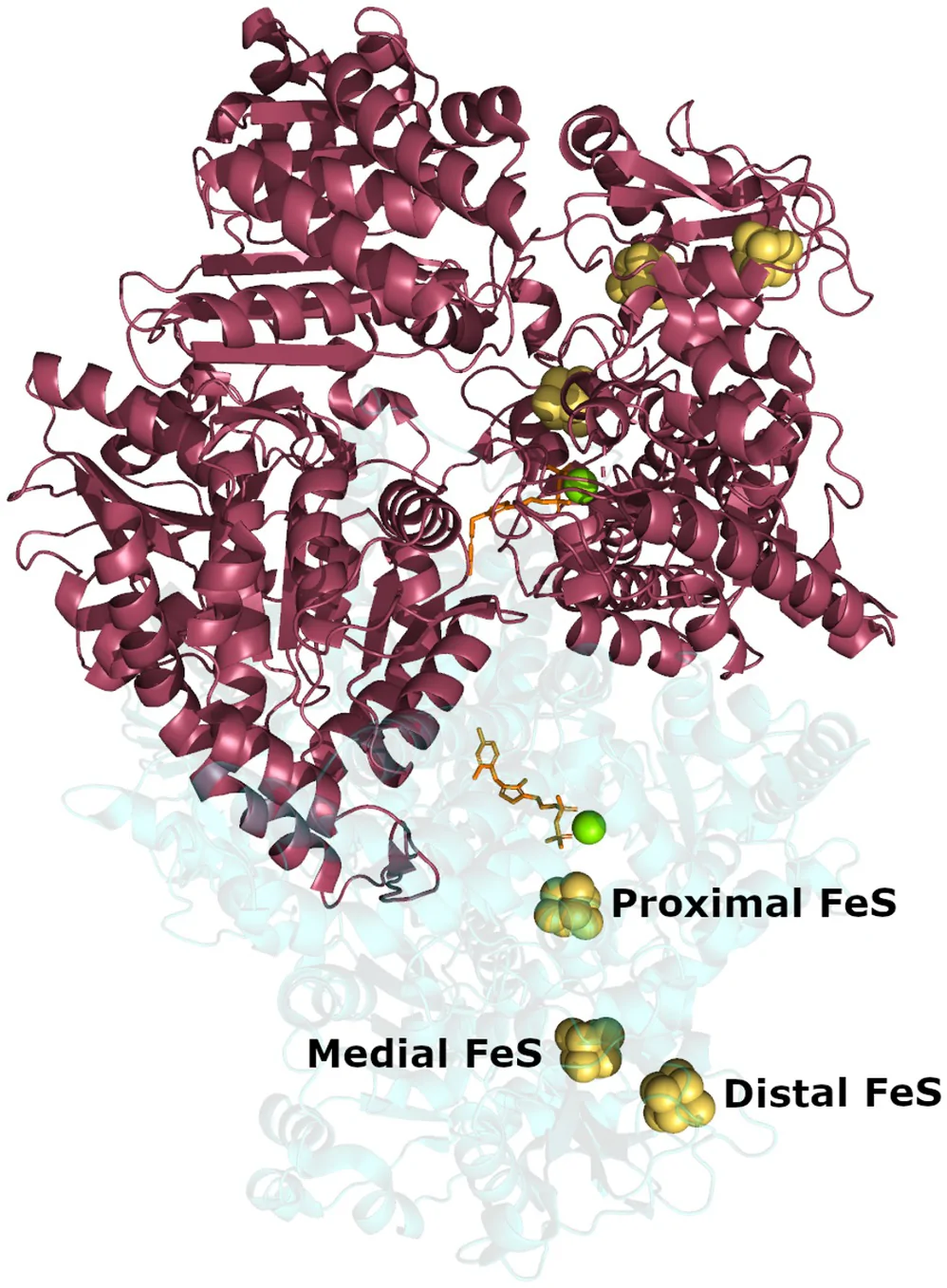
3D structure of the homodimeric PFOR of *N. thermoacetica* (PDB 6CIN) (Chen et al., 2018). FeS clusters are represented in yellow balls, Mg^2+^ ion in green ball and TPP cofactor in orange sticks. The two monomers are shown in two different colors.

The catalytic mechanism of PFOR is well characterized (Figure 2a) and supported by, among other data, crystallographic and cryo‐EM structures (from *D. africanus* and *N. thermoacetica* PFOR), either in the presence or absence of one of the enzyme's substrates (pyruvate or CoA) (Cavazza et al., 2006; Chabrière et al., 1999; Charon et al., 1999a; Chen et al., 2018). These structures have revealed multiple catalytic states which are characterized by various TPP derivatives as intermediates. They have also highlighted the binding sites of the substrates within the enzyme and the conformational changes that occur during catalysis.

**FIGURE 2:**
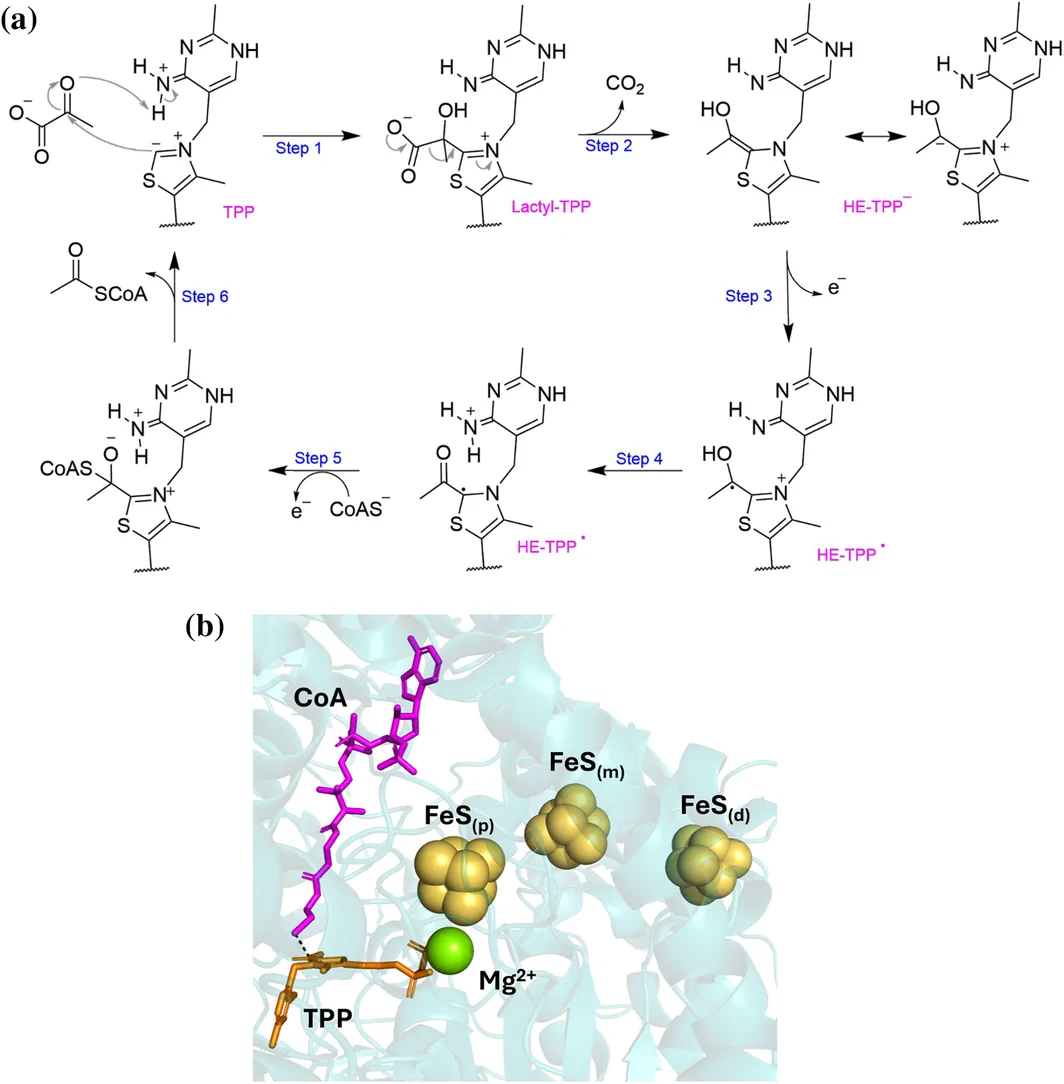
PFOR reaction mechanism. (a) Catalytic mechanism of PFOR in the oxidation direction (Adapted from: Bonitatibus et al., 2025; Chen et al., 2018; Ragsdale, 2003). See the text for the names of the intermediates. (b) Superposition of the HE‐TPP^•^ intermediate from *N. thermoacetica* PFOR obtained in the absence of CoA (PDB 6CIP) onto the structure determined in the presence of CoA (PDB 6CIQ). The black dotted line indicates the distance (2.3 Å) between the sulfur atom of CoA and the carbon atom of the TPP^•^ acetyl group.

The reaction cycle begins with the nucleophilic addition of the TPP ylide on the carbonyl carbon of pyruvate (Figure 2a, step 1). This addition is accompanied by protonation of the substrate oxygen from the positively charged thiazolium nitrogen of TPP, forming the covalent lactyl‐TPP intermediate (PDB 2C3P and PDB 6CIO). This intermediate then undergoes decarboxylation to yield the two mesomeric forms of hydroxyethyl‐TPP anion (HE‐TPP^−^) (step 2). A first electron is transferred to the enzyme's FeS clusters, generating the hydroxyethylidene‐TPP radical (HE‐TPP^•^), a highly unstable intermediate (step 3) (Menon & Ragsdale, 1997). The radical undergoes rearrangement to form a stabilized carbonyl radical species in the absence of CoA (step 4) (PBD 2C3Y (Cavazza et al., 2006) and PDB 6CIP (Chen et al., 2018)), which has been characterized by electron paramagnetic resonance (EPR) spectroscopy (Cammack et al., 1980; Kerscher & Oesterhelt, 1981a; Mansoorabadi et al., 2006; Ragsdale, 2003). In step 5, CoA binding occurs, which transforms a relatively active open site (determined by the position of domain III) to a closed configuration, moving an average of 7 Å (Chen et al., 2018), and positioning the thiolate group of CoA toward the HE‐TPP^•^ radical via hydrogen bonding and electrostatic interactions with the pyrophosphate group of CoA (Figure 2b). Through this process, a 10,000‐fold rate of acceleration of the decay of the HE‐TPP radical occurs (Furdui & Ragsdale, 2000). The 2.3 Å distance between the sulfur atom of CoA and the carbon atom of the TPP^•^ acetyl group favors a nucleophilic attack of the thiolate on the TPP acetyl carbon. The final step consists of CoA acetylation, yielding acetyl‐CoA, followed by product release (step 6), restoring the active site to its initial state for a new catalytic cycle.

A tentative catalytic mechanism has been proposed for the bifunctional PFORs, in which CoA has only a structural role in the PDC reaction. The first two steps of the cycle were suggested to be common to the two reactions. A “switch” mechanism was suggested to occur later on to give either acetyl‐CoA or acetaldehyde (Eram & Ma, 2013; Ma et al., 1997).

The structure resolution of *D. africanus* PFOR as well as mutagenesis studies have revealed the mechanism of its air‐resistance: both the formation of a disulfide bridge which is associated with a conformational change and a methionine (Met1203 in *D. africanus* or a histidine in other sulfate‐reducing bacteria PFOR) acting as an oxidant scavenger protect the proximal FeS cluster against O_2_ damage (Vita et al., 2008).

## 3 SUBUNIT AND DOMAIN COMPOSITION DIVERSITY AND OLIGOMERIZATION

As shown in Table 1, the PFORs identified so far are primarily composed of one (α or α′) or four (α𝛽𝛾𝛿) subunits. A few consist of either two (α𝛽 or α𝛽′) or five (α𝛽𝛾𝛿ε) subunits. In this review, PFORs are referred to as “one‐subunit”, “two‐subunit”, “four‐subunit” or “five‐subunit” enzymes, even though they have a dimeric quaternary organization (see end of this section). Structures of one‐subunit PFORs have revealed up to seven domains, named I to VII (Figure 3a).

**FIGURE 3:**
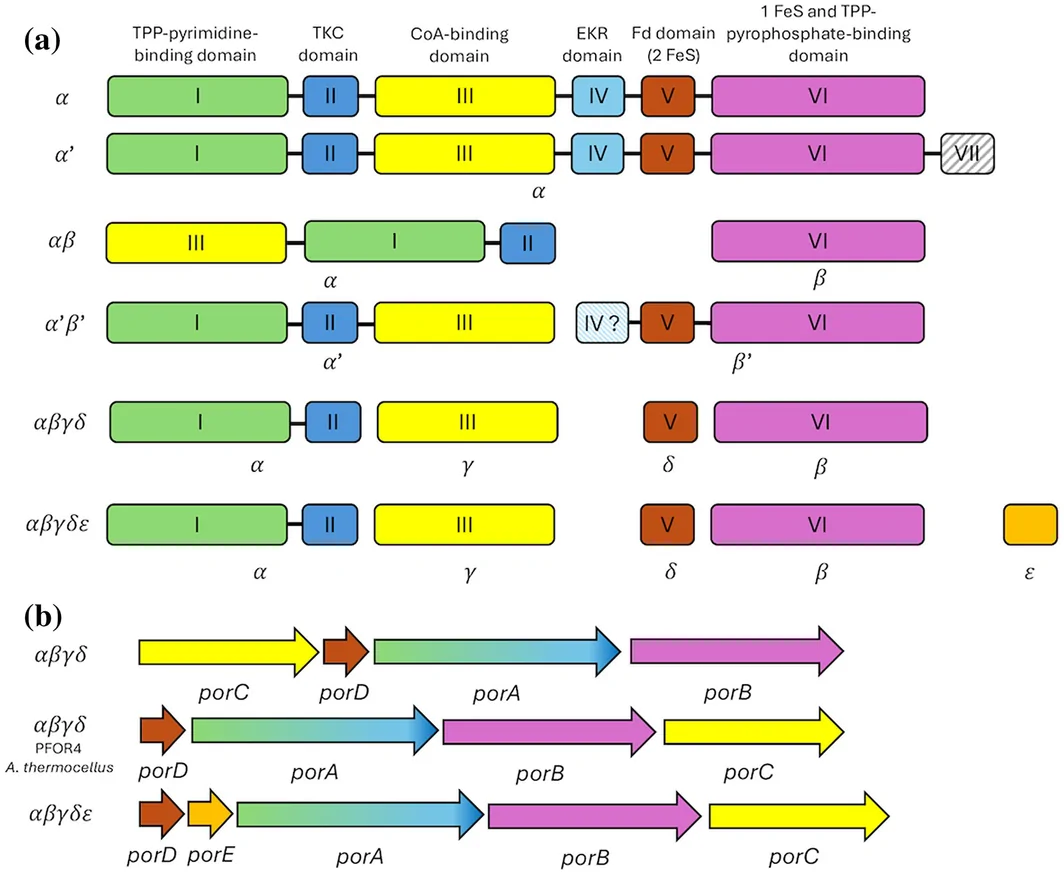
PFORs subunits and domains arrangement. (a) Subunit and domain composition of PFORs, based on amino acid sequences, and structural and biochemical data of enzymes listed in Table 1. The PFOR domains are annotated according to the Uniprot prediction (The UniProt Consortium et al., 2025) and the InterPro domain attribution (Blum et al., 2025). TKC domain: Transketolase domain, Fd domain: Ferredoxin‐type domain. (b) Organization of the gene clusters that encode α𝛽𝛾𝛿‐PFORs, PFOR4 from *A. thermocellus* and α𝛽𝛾𝛿ε‐PFORs. Genes and their corresponding subunits (or domains) are represented by the same color.

All PFORs contain domains I, II, III and VI, essential for the enzyme's activity. Domains I and VI house binding sites for TPP and pyruvate, domain III binds CoA, and domain VI binds the proximal FeS cluster (Figures 2b and 3a). Domain II, present in all PFORs, does not appear to have a role in any cofactor or substrate binding. Domain IV, present in the one‐subunit PFORs, is a linker domain characterized by a motif composed of the three amino acids EKR (Chen et al., 2018; Cossu et al., 2024; Katsyv, Schoelmerich, et al., 2021). Domain V contains the residues required for binding the medial and distal FeS clusters. Two‐subunit (α𝛽) PFORs lack domain V, and therefore possess only one FeS cluster (Witt et al., 2019). Domain VII is only present in most one‐subunit PFORs from sulfate‐reducing bacteria (Table 1). This domain contains the disulfide bridge that protects PFORs from damage by O_2_ in *D. africanus* (Charon et al., 1999b; Pieulle et al., 1997; Witt et al., 2019) and possibly in *Nv*H (Pieulle et al., 2011; Ramel et al., 2015) and other sulfate‐reducing bacteria (Vita et al., 2008). For these latter ones, experimental validation is required.

Sequence analysis and domain assignment revealed that the α subunit of the two‐subunit (α𝛽) PFORs has a different domain organization than all other PFORs, with domain III located at the N‐terminus of domain I (Figure 3a). This configuration has only been previously described for OGOR and OFOR (Chen et al., 2019; Cossu et al., 2024; Gibson et al., 2016), but not for PFOR. Nevertheless, α𝛽‐PFORs, all of which are derived from Archaea, can use various 2‐oxoacids as substrates in addition to pyruvate (see Section 5 and Table 1).

The two‐subunit (α′𝛽′) PFOR from the bacterium *Magnetococcus marinus* has a distinct domain arrangement compared to two‐subunit (α𝛽) PFORs. Bonitatibus et al. proposed that domain IV is present in the 𝛽′ subunit of this enzyme (Bonitatibus et al., 2025). However, we could not identify an EKR signature in its sequence (accession number ABK44249). This PFOR is the only one of its kind that has been characterized to date.

Sequence analysis of α𝛽𝛾𝛿ε‐PFORs shows that ε subunit does not seem to correspond to any of the seven domains (Gibson et al., 2016). This subunit is named PorD in *Aquifex aeolicus* (Prioretti et al., 2023) and *Hydrogenobacter thermophilus* (Ikeda et al., 2006) PFORs although it does not correspond to PorD (𝛿) of α𝛽𝛾𝛿‐PFORs. As there is no 3D structure available for five‐subunit PFORs, the role and positioning of the ε subunit in the enzyme remain unknown.

In this review, the PFOR from the methanogenic archaeon *Methanococcus maripaludis* is regarded as an α𝛽𝛾𝛿‐PFOR (Table 1), despite having been characterized as a five‐subunit enzyme (Lin et al., 2003; Lin & Whitman, 2004). The fifth subunit, called PorE, is not always present in the purified enzyme. It is a polyferredoxin with no similarity to the extra subunit found in the commonly accepted five‐subunit PFORs (Porat et al., 2006). The PFOR gene cluster from *Methanothermobacter marburgensis* also contains the gene encoding the polyferredoxin. However, the PFOR of this archaeon is a four‐subunit enzyme (Tersteegen et al., 1997), which suggests that this protein is likely not a subunit of the complex in *M. maripaludis*. A complete understanding of PFOR (and OFOR) subunit composition is still a long way off, even at the genetic level.

Figure 3a shows that one‐subunit PFORs resemble a concatenation of the four subunits of α𝛽𝛾𝛿‐PFORs in the order α‐𝛾‐𝛿‐𝛽. However, the genes of most four‐subunit PFORs are organized in the order *porC‐porD‐porA‐porB* (except for PFOR4 from *Acetivibrio thermocellus* and for the five‐subunit PFORs which have a similar gene order [*porD‐porA‐porB‐porC* and *porD‐porE‐porA‐porB‐porC*, respectively]) (Figure 3b). These observations support the hypothesis proposed earlier that a fusion occurred during the evolution of this enzyme family through several recombination and fusion events to produce either one‐ or two‐subunit PFORs (Kletzin & Adams, 1996; Zhang et al., 1996).

All OFOR structures show that the subunits form a similar core that dimerizes, regardless of their number (Chabrière et al., 1999; Chen et al., 2018, 2019; Cossu et al., 2024; Garczarek et al., 2007; Gibson et al., 2015; Yan et al., 2016). The formation of this dimer (of a “monomeric” core comprising one, two, four, or five subunits) is supported by experimental evidence (structures, gel filtration, or sedimentation analyses, or non‐denaturing gels) for more than a third of the characterized PFORs (Table 1). Notably, the bacterial *Nv*H PFOR has been characterized as a homooctameric (tetramer of dimer) enzyme exceeding 1000 kDa (Garczarek et al., 2007).

## 4 DISTRIBUTION OF PFORs ACROSS DOMAINS OF LIFE

PFORs are found in all three domains of life, although their occurrence is limited in eukaryotes (Duwor et al., 2024). The subunit organization of PFORs that have been characterized so far shows a strong taxonomic domain bias: archaeal PFORs are exclusively two‐ or four‐subunit complexes, bacterial enzymes are predominantly one‐ or four‐subunit forms (with exceptions of five‐subunit enzymes and the only example of the α′𝛽′ from *M. marinus*). All known eukaryotic representatives belong to the one‐subunit type (Table 1).

Figure 4 shows a phylogenetic tree constructed using MEGA12 software (Hall, 2013; Kumar et al., 2024), from concatenated subunit sequences from enzymes listed in Table 1. This tree reveals that PFORs cluster primarily according to subunit composition rather than to taxonomy, with the two‐subunit α′𝛽′ enzyme from *M. marinus* forming a distinct branch relative to other two‐subunit PFORs. One‐subunit bacterial and eukaryotic enzymes cluster within a single large clade, whereas archaeal PFORs are partitioned into two clades that span two‐ and four‐subunit organizations. Except for *A. thermocellus* PFOR4, bacterial and archaeal four‐subunit enzymes are grouped into a single clade. *A. thermocellus* PFOR4 appears to be closely related to the α𝛽𝛾𝛿ε‐PFORs, and they all share a similar gene organization, as mentioned above (Section 3, Figure 3b). As previously found, bacterial one‐subunit PFORs separate into two sub‐clusters (Gibson et al., 2016; Horner et al., 1999; Rotte et al., 2001) and eukaryotic PFORs form a third sub‐cluster (Duwor et al., 2024). The clear segregation of PFORs with different subunit compositions underscores the fact that variations in subunit makeup are strongly lineage‐dependent. This variation in architecture may not be neutral, but rather reflect functional adaptations to distinct lifestyles, physiological constraints or environmental factors, such as living in the presence or absence of oxygen (Figure 4) or at extreme temperatures. These differences in subunit composition may “optimize” the PFOR and confer a functional advantage in the specific physiological context of each lineage. This pattern suggests that evolution has proceeded mainly through structural rearrangements of a conserved TPP/FeS core, allowing the same catalytic module to be repeatedly redeployed into diverse physiological and ecological contexts across Bacteria, Archaea and Eukarya. This tree topology supports a complex evolutionary history for PFOR involving multiple events and horizontal gene transfer between lineages (Duwor et al., 2024; Horner et al., 1999; Rotte et al., 2001).

**FIGURE 4:**
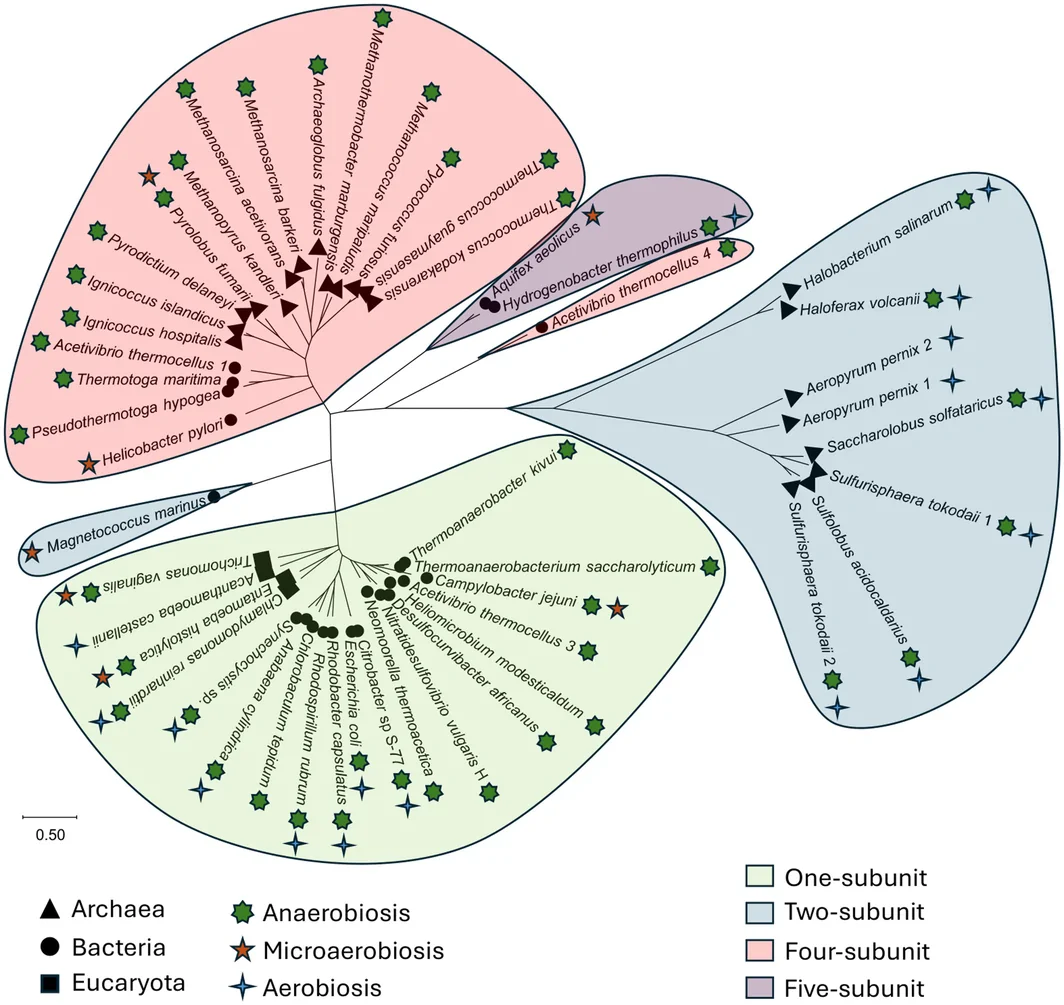
Phylogenetic tree of enzymes exhibiting PFOR activity. For the multi‐subunit PFORs, complete protein sequences (except for the first methionine which was removed) of all subunits were fused (in the same order as in Figure 3a) to establish a more accurate phylogeny, except for the ε subunit of the five‐subunit PFORs which was omitted. Accession numbers of the α‐subunit sequences are indicated in Table 1. The tree was made using MEGA12 software, with 1000 rounds of replication. The colors indicate the number of subunits in the PFOR, the black symbols indicate domains of life, and the star symbols indicate the O_2_ requirements for organism growth. The scale‐bar represents the amount of evolutionary change (substitutions per amino‐acids site). For clarity, the bootstrap values have been omitted from this tree, but a different representation including these values is shown in Figure S1.

## 5 SUBSTRATE DIVERSITY: 2‐OXOACIDS, SMALL REDOX ACTIVE PARTNERS AND PROTEIN‐BASED REDOX CARRIERS

### 5.1 2‐Oxoacids

In vitro, PFORs collectively display a broad but uneven substrate range toward 2‐oxoacids, as illustrated in Table 1. All characterized PFORs oxidize pyruvate (with *K*_m_ ranging from 9.3 µM to 14.5 mM (Table S1)). When tested with other substrates, many of them accept 2‐oxobutyrate, consistent with the minimal structural difference between these two substrates (Figure 5). Several enzymes also convert 2‐oxaloacetate, although this could be an artifact resulting from its decomposition into pyruvate (Kunow et al., 1995; Yu & Sivitz, 2020). Most archaeal two‐subunit PFORs also use 2‐oxoglutarate as substrate, the typical substrate of OGOR, blurring the functional boundary with canonical OGOR. In vitro, a few archaeal and eukaryotic PFORs can catalyze the oxidation of at least two other substrates, with specific activities higher than 30% of the specific activity of pyruvate oxidation. When measured, the catalytic efficiency with these alternative substrates is lower than with pyruvate (Kerscher & Oesterhelt, 1981b; Kunow et al., 1995; Lin et al., 2003; Nishizawa et al., 2005; Pineda et al., 2010). Nevertheless, this suggests that some members of the family should be better classified as OFORs rather than strictly “pyruvate‐specific” enzymes. By contrast, a wider panel of 2‐oxoacids (including aromatic or dicarboxylic 2‐oxoacids) generally supports only low activity. As an exception, *Aeropyrum pernix* PFORs efficiently oxidize five or more of these non‐pyruvate substrates with a specific activity of at least 35% that of pyruvate (Nishizawa et al., 2005) and thus exemplifies genuine OFOR‐like behavior. In summary, some PFORs appear to be strictly “pyruvate specific” (Tables 1 and S1), whereas others can efficiently oxidize multiple 2‐oxoacids and should therefore be more accurately categorized as OFORs. The boundary between these categories remains ill‐defined in the literature (Gibson et al., 2016), and substrate profiles determined in vitro are unlikely to capture the full range, or true physiological relevance, of the substrates used in vivo.

**FIGURE 5:**
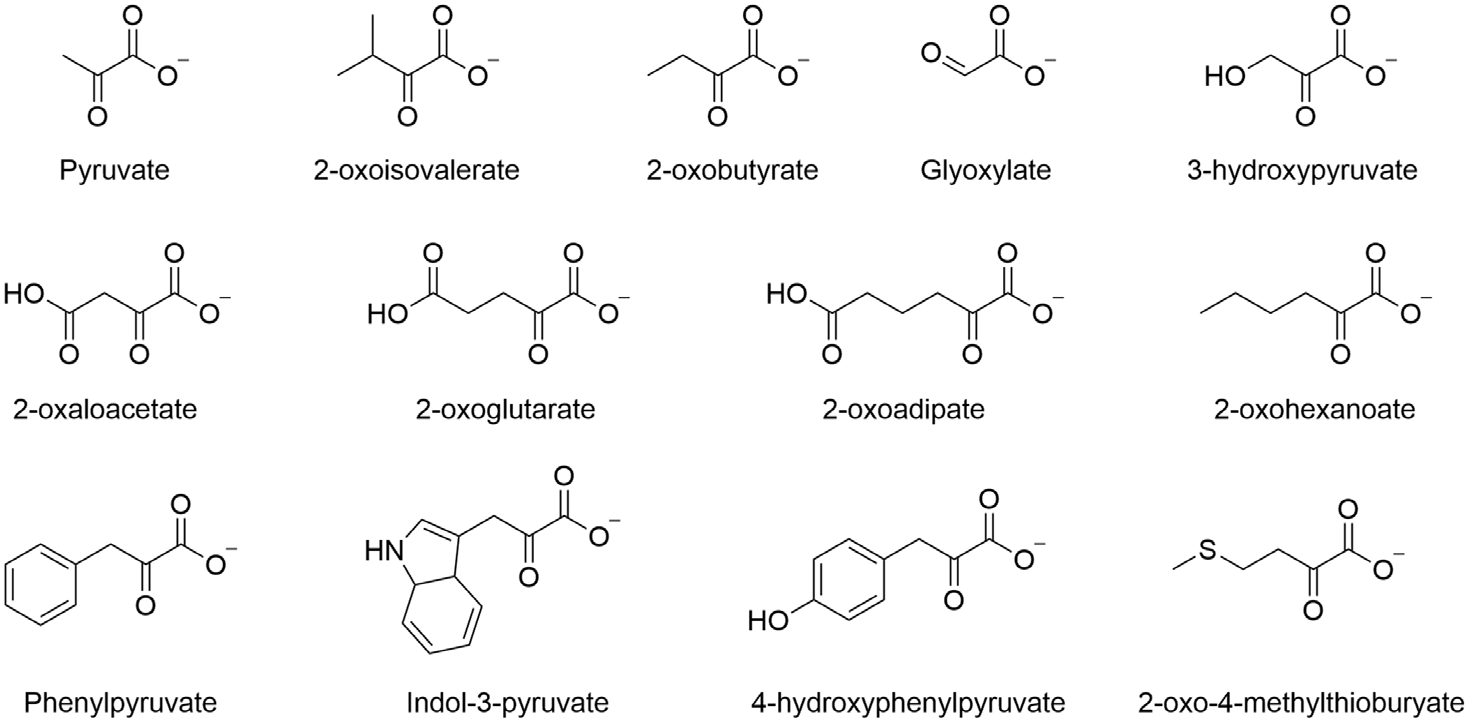
Structure of the 2‐oxoacids used, in vitro, as substrates by PFORs (as shown in Table 1).

### 5.2 Artificial electron acceptors and donors

PFOR activity is commonly monitored by coupling pyruvate oxidation to reduction of chemical compounds, typically methyl viologen (MV) (*E*° ≈ −440 mV) or benzyl viologen (*E*° ≈ −360 mV) (Mansfield, 1960) (Table S1). Flavin‐based compounds (FMN, FAD and riboflavin (*E*°′ ≈ −220 mV) (Lowe & Clark, 1956)), have also been employed as electron acceptors, as have other redox dyes such as Nitro blue tetrazolium, Phenosafranin, Janus Green B, Nitazoxanide, Triphenyl tetrazolium chloride and Methylene blue (*E*°′ from −360 to 11 mV (Jámbor, 1954; Mansfield, 1960; Sisson et al., 2002; Viseu et al., 1990)) (Note: reduction potentials are given vs. standard hydrogen electrode and apparent *E*°′ are given at pH 7). The apparent potential *E*°′ of the acetyl‐CoA/CO_2_ to pyruvate/CoA reaction has been estimated to range from *E*°′ = −515 mV (Witt et al., 2019) to −540 mV (Menon & Ragsdale, 1997). The oxidation of pyruvate by these artificial acceptors is thermodynamically favorable considering standard redox potentials and even more favorable considering Nernst potentials (that take experimental conditions into account). Conversely, in the reverse (CO_2_‐reducing) direction, only reduced low‐potential Fd, reduced flavodoxin (Fld) or reduced MV have been employed as electron donors (Blaschkowski et al., 1982; Bonitatibus et al., 2025; Furdui & Ragsdale, 2000; Ikeda et al., 2010; Li & Elliott, 2016; Li, Steindel, et al., 2021; St. Maurice et al., 2007; Witt et al., 2019; Xiao et al., 2021). In this case, the reaction is thermodynamically favorable only when Nernst potentials are considered (Witt et al., 2019).

Overall, the redox‐active chemicals spanning a broad range of standard potentials can substitute for physiological partners in vitro, providing convenient spectrophotometric readouts while underscoring that true thermodynamic feasibility and driving force must be interpreted in the context of Nernstian rather than standard potentials alone.

### 5.3 Potential physiological redox partners of PFOR

For roughly one‐third of the PFORs listed in Table 1, either no candidate physiological redox partner has been tested, or activity has only been measured in crude extracts, precluding a clear assignment (Table S1). When tested, PFORs are active with Fd. Several PFORs also reduce Flds and/or rubredoxins (Rd), sometimes more efficiently than Fd. Fds are small FeS proteins that typically contain one or two clusters (plant‐type [2Fe‐2S] vs. bacterial‐type [4Fe‐4S] and/or [3Fe‐4S]) and span a broad range of redox potentials from about −700 to −100 mV (Hosseinzadeh & Lu, 2016). Fld harbors FMN with potential for its two redox transitions of −92 to −245 mV and −370 to −515 mV (Sykes & Rogers, 1984). Rd contains a four‐cysteine coordinated iron with redox potentials typically between −50 and +50 mV (Hosseinzadeh & Lu, 2016). The reduction of these proteins by PFORs suggests that they have a high degree of flexibility in terms of the partner proteins and cofactor they contain.

The *Escherichia coli* PFOR, for example, behaves as a pyruvate:flavodoxin/ferredoxin oxidoreductase in vitro, reducing an *E. coli* Fld as well as the *E. coli* [2Fe‐2S] Fd and the *Clostridium pasteurianum* [4Fe‐4S] Fd (Blaschkowski et al., 1982). In vivo, the *C. pasteurianum* Fd and the *E. coli* FldA and FldB were shown to be reduced by this PFOR (Akhtar & Jones, 2009). Fld also appears to be the partner for PFORs from *Klebsiella pneumoniae, Helicobacter pylori, Campylobacter jejuni*, *Rhodobacter capsulatus, Nostoc* sp., *D. africanus* and *Nitratidesulfovibrio vulgaris* Miyazaki, even if it is frequently in addition to Fd (Alqurashi et al., 2021; Hughes et al., 1995; Kendall et al., 2014; Ogata & Yagi, 1986; Pieulle et al., 1995; Razquin et al., 1995; Wahl & Orme‐Johnson, 1987; Yakunin & Hallenbeck, 1998) (Table S1).

Rd has been found to be an efficient partner in pyruvate oxidation (Furdui & Ragsdale, 2000; Lin et al., 2003; Pieulle et al., 1995; Yoon et al., 1999) but was unable to act as an electron donor in pyruvate synthesis, probably due to its higher redox potential (Furdui & Ragsdale, 2000; Yoon et al., 1999). The PFOR from *M. maripaludis* differs from other PFORs in that it exhibits much higher activity with Rd than with Fd for pyruvate oxidation (Lin et al., 2003).

The redox and structural properties of the mono‐ and dicluster partner Fd are both significant for PFOR activity (Bonitatibus et al., 2024; Katsyv, Schoelmerich, et al., 2021; Li, Steindel, et al., 2021). Studies have shown that the redox potential of Fd is crucial in determining the direction of the reaction and whether a PFOR (or, more generally, an OFOR) can reduce CO_2_ in vitro, regardless the enzyme's physiological role (Li & Elliott, 2016; Witt et al., 2019). Additionally, modulating the Fd redox potential through engineering offers a means to precisely adjust PFOR catalysis (Li, Steindel, et al., 2021).

Overall, the data suggest that PFORs can interact with a variety of small redox proteins that differ in terms of their potential ranges and structural classes. Additionally, their apparent partner specificity in vitro appears to be relatively weak, although this does not exclude the existence of PFOR's selectivity and preference for a specific Fd (Bonitatibus et al., 2024, 2025; Katsyv et al., 2023).

## 6 DIVERSITY OF BACTERIAL AND ARCHAEAL METABOLIC PATHWAYS INVOLVING PFOR

PFOR is a pivotal enzyme that fulfills several key physiological roles associated with the “pyruvate node”, thus linking carbon metabolism to cellular redox balance in many anaerobic and microaerophilic organisms (Figure 6). The specific roles played by PFORs depend on the direction of the reaction (oxidation or reduction), the type of metabolism (heterotrophic or autotrophic) and whether the environment is oxygenated or anoxic. This enzyme can either participate in the oxidation of pyruvate in heterotrophic organisms, or it can catalyze the reverse reaction in autotrophic organisms, fixing CO_2_ and forming pyruvate. This is the case in a wide variety of organisms (Figure 4) with respiratory (e.g., *H. pylori* or *A. aeolicus*) (Hughes et al., 1998; Prioretti et al., 2023; St. Maurice et al., 2007), fermentative (e.g., *Clostridium* sp. or *Thermotoga maritima*) (Blamey & Adams, 1994; Kletzin & Adams, 1996; Meinecke et al., 1989), methanogenic (e.g., *Methanosarcina*) (Bock et al., 1996; Cossu et al., 2024), acetogenic (e.g., *Thermoanaerobacter kivui* or *N. thermoacetica*) (Chen et al., 2018; Islam et al., 2015; Katsyv, Schoelmerich, et al., 2021) or photosynthetic (e.g., *Synechocystis*, *R. capsulatus* or *Chlorobaculum tepidum*) (Feng et al., 2010; Gutekunst et al., 2014; Yakunin & Hallenbeck, 1998; Yoon et al., 1999) capabilities.

**FIGURE 6:**
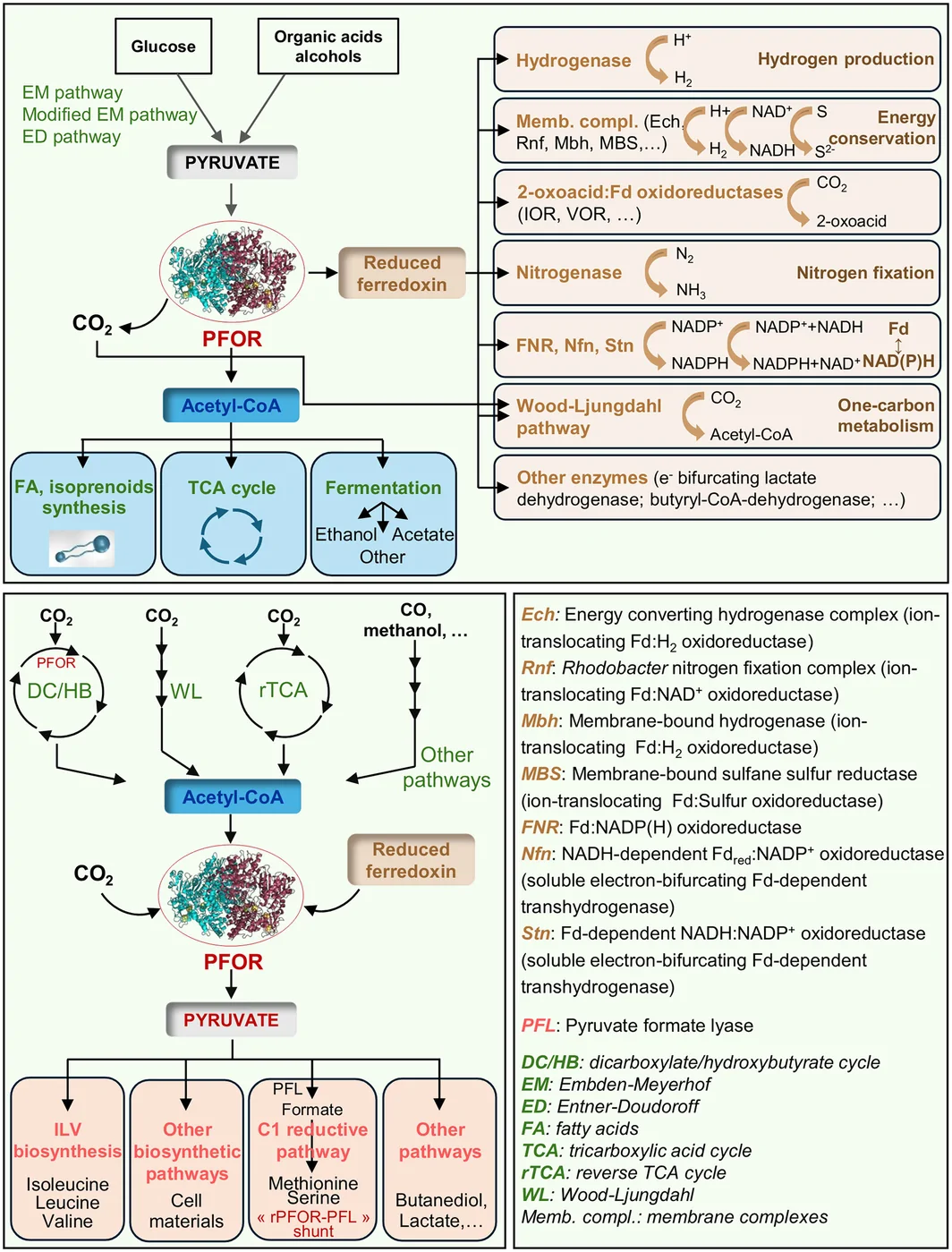
Bacterial and archaeal metabolic pathways involving PFOR. (a) for the top panel, PFOR working in the decarboxylation of pyruvate. (b) for the bottom left panel, PFOR working in the carboxylation direction.

### 6.1 In the direction of pyruvate decarboxylation

PFORs functionally replace the pyruvate dehydrogenase (PDH) complex, which operates under aerobic conditions. However, they reduce a low‐potential electron acceptor (Fd or Fld in most cases) rather than NAD^+^, significantly impacting the way in which redox energy is managed (Chen et al., 2022; Ludwig & Bryant, 2011; Wang et al., 2022).

Pyruvate is produced in heterotrophic organisms that feed on glucose, as well as carbohydrates, peptides, and organic acids (Figure 6a). Glucose is metabolized via the Embden–Meyerhof (EM) or the Entner–Doudoroff (ED) pathways, both of which release pyruvate (Eram et al., 2015; Foulquier et al., 2022; Huang et al., 2012; Li, Ye, et al., 2021; Ludwig & Bryant, 2011; Verhees et al., 2003). PFOR then converts the produced pyruvate into acetyl‐CoA and CO_2_.

Not only is acetyl‐CoA involved in several essential biosynthetic processes, such as the production of fatty acids (Alqurashi et al., 2021; Hocking et al., 2014; Li, Ye, et al., 2021; Schübbe et al., 2009), but it can also be further metabolized for energy conservation generating a variety of products depending on the organism and growth conditions. In many cases, acetate and ATP are released from acetyl‐CoA in both fermentative and respiratory organisms (Flowers et al., 2018; Gutekunst et al., 2014; Huang et al., 2012; Hughes et al., 1998; Kanai et al., 2015; McNeely et al., 2011; Santiago‐Martínez et al., 2016). Ethanol and other end products (e.g., butyrate, butanol, and acetone) can also be produced by many anaerobic fermentative microorganisms (Chen et al., 2022; Cui et al., 2019; Eram et al., 2015; Foulquier et al., 2022; Keller et al., 2017; Pavao et al., 2023; Payne et al., 2022). Acetyl‐CoA released by PFOR catalysis can also feed the tricarboxylic acid (TCA) cycle, yielding energy, precursors for biosynthesis, as well as reducing power in the form of NAD(P)H, quinol or reduced Fds (Hocking et al., 2014; Verhees et al., 2003; Wang et al., 2022).

PFORs are also crucial in supplying highly energy‐rich reducing equivalents in the form of low‐potential Fds (or alternatively Flds), which can fuel numerous enzymes in anaerobic organisms. These electron carriers supply electrons to hydrogenases (catalyzing the reversible conversion of protons into H_2_) either to discharge the reducing power during fermentation (H_2_ production) (Foulquier et al., 2022; Gutekunst et al., 2014; Pavao et al., 2023), or for energy conservation. This occurs either via the mechanism of electron bifurcation as in the case of the soluble hydrogenase HydABC from *T. maritima* (Furlan et al., 2022; Liang et al., 2022), or via a transmembrane electrochemical ion gradient as with membrane‐bound hydrogenases Ech and Mbh (enzyme full names are listed in Figure 6) (Baum et al., 2024; Kanai et al., 2015; Meuer et al., 2002; Payne et al., 2022). Other membrane respiratory complexes that regenerate Fds include Rnf (which releases NADH) (Biegel et al., 2009; Cui et al., 2019; Liang et al., 2022; Pavao et al., 2023), MBS (which reduces sulfur) (Liang et al., 2022) and other complex I‐like enzymes (Alqurashi et al., 2021). A wide range of other enzymes are fueled by reduced Fds, including potentially some OFORs, such as IORs and VORs (Mwirichia et al., 2016); nitrogenases, which reduce N_2_ to NH_3_ in diazotrophs (Chen et al., 2022; Neuer & Bothe, 1982; Poudel et al., 2018; Razquin et al., 1995; Yakunin & Hallenbeck, 1998); and enzymes linking the pool of reduced Fds to the NAD(P)H pool, such as the Fd:NADPH oxidoreductase (FNR, Fpr, FNO, and Fnor) and the electron‐bifurcating, NADH‐dependent Fd:NADP transhydrogenases, Nfn and Stn (Cui et al., 2019; Foulquier et al., 2022; Huang et al., 2012; Hughes et al., 1998; Katsyv, Jain, et al., 2021; Kumar et al., 2023; Li, Ye, et al., 2021; Liang et al., 2022; McNeely et al., 2011; Wang et al., 2022). These modules convert the reducing power of Fds into NADH/NADPH, and vice versa, establishing PFOR as the gateway to a vast redox network. In some microorganisms, PFOR connects central carbon metabolism to one‐carbon (C1) metabolism, with reduced Fds feeding electrons to enzymes in the Wood–Ljungdahl (WL) pathway for CO_2_ reduction under certain conditions. Additionally, this pathway can re‐assimilate CO_2_ produced directly by PFOR (Huang et al., 2012; Pavao et al., 2023; Ragsdale, 2008).

In summary, PFOR acts as a link between glycolysis (or other pathways of glucose degradation) and pathways derived from acetyl‐CoA, including fermentation and biosynthetic processes. PFOR contributes to the energy metabolism and assimilation pathways of many organisms by supplying electrons with very low redox potentials. PFOR also connects central metabolism to C1 metabolism.

### 6.2 In the direction of pyruvate synthesis

The fundamental process of CO_2_ fixation into organic compounds occurs through various pathways in autotrophic bacteria and archaea. In anaerobiosis, all these metabolic routes rely on the formation of pyruvate, needed for anabolic purposes. This formation is catalyzed by reductive carboxylation of acetyl‐CoA by PFOR, also known as pyruvate synthase. PFOR is a CO_2_‐fixing enzyme that plays a role in the reductive TCA (rTCA) cycle, the dicarboxylate/hydroxybutyrate (DC/HB) cycle, and the WL pathway (Figure 6b). It assimilates released acetyl‐CoA, derived from these pathways, into pyruvate which is then converted into intermediates for cell carbon molecules synthesis (Berg et al., 2010; Feng et al., 2010; Fuchs, 2011; Furdui & Ragsdale, 2000; Ikeda et al., 2010; Meuer et al., 2002). In the photosynthetic bacterium *Rhodospirillum rubrum*, pyruvate is formed directly by PFOR from acetyl‐CoA derived from assimilated acetate and feeds the Isoleucine‐Leucine‐Valine biosynthetic pathway (Leroy et al., 2015) (Figure 6b). *A. thermocellus*, a bacterium that degrades cellulose through fermentation, can also assimilate CO_2_ via a reductive C1 metabolic pathway, contributing to methionine and serine synthesis (Xiong et al., 2016). Together with pyruvate formate lyase (PFL), PFOR works in reverse to convert CO_2_ into formate, which then enters the one‐carbon pathway (Xiong et al., 2016) (Figure 6b).

Carboxylation of acetyl‐CoA by PFOR is challenging because the reaction must overcome a significant energy barrier. It requires Fd, acting as a highly effective reductant. In phototrophs such as *Chlorobaculum*, Fd is reduced by the photosynthetic chain (Feng et al., 2010; Walters et al., 2024). In contrast, in chemolithoautotrophic organisms, this process may be catalyzed by an electron‐bifurcating enzyme. These latter organisms generally grow using energy sources with a higher redox potential than Fd. These particular enzymes solve this energetic problem by coupling an endergonic reaction (Fd reduction) to an exergonic reaction (reduction of a second acceptor with a higher potential) (Fuchs, 2011; Müller et al., 2018). The unfavorable reduction of Fd that fuels PFOR is also achieved by membrane‐bound complexes at the expense of the ion electrochemical gradient (Fuchs, 2011). One example is Ech in *Methanosarcina barkeri* (Meuer et al., 2002).

### 6.3 In vivo reversibility

Depending on the growth conditions, a PFOR can function in both directions within the same organism. In *R. rubrum*, the PFOR has been proposed to play a role in both acetate assimilation (working in pyruvate formation) and in nitrogen fixation (working in pyruvate oxidation under dark anaerobic conditions) by providing the driving force for nitrogenase activity in the form of reduced Fds (Edgren & Nordlund, 2006; Leroy et al., 2015). Pyruvate consumption and synthesis by PFOR have also been demonstrated in methanogenic archaeal *Methanosarcina* species (López Muñoz et al., 2015; Richter et al., 2024). Depending on its nutritional status, *M. acetivorans* either generates pyruvate using PFOR for glycogen synthesis as an energy source or decarboxylates pyruvate during glycogen degradation, releasing acetyl‐CoA that is converted to methane. The concentration of CO_2_ and the ratio of reduced to oxidized Fd were suggested to play a role in determining the direction of the reaction (Santiago‐Martínez et al., 2016). PFOR has been proposed to function in both directions in *N. thermoacetica* (Islam et al., 2015) and in *M. marinus* (Schübbe et al., 2009), bacteria that can grow both heterotrophically and autotrophically.

### 6.4 Multiplicity of PFOR genes within a single organism

Some microorganisms possess multiple genes that encode PFORs (Zhou et al., 2015). *T. kivui* encodes two highly similar PFORs, but only one appears to be produced under heterotrophic conditions on glucose (Katsyv, Schoelmerich, et al., 2021). Likewise, the two PFORs of the archaeon *S. tokodaii* str. 7 are similar in sequence but are probably not produced under the same growth conditions (Yan et al., 2016). In *A. thermocellus*, two out of the four PFORs act as pyruvate synthase (Xiong et al., 2016). Two PFORs (68% identical), both of whose genes were shown to be transcribed, occur in the filamentous, multicellular, N_2_‐fixing cyanobacterium *Anabaena* sp. PCC 7120 (Schmitz et al., 2001).

### 6.5 Role in the presence of oxygen and in redox control

Several PFORs were isolated from archaea with an aerobic lifestyle and have been shown to be stable in the presence of O_2_. This is the case for PFORs from *Sulfolobus*, *Saccharolobus* and *Sulfurisphaera* species, and *A. pernix* (Alharbi et al., 2022; Nishizawa et al., 2005; Witt et al., 2019; Yan et al., 2016). The same is true for the PFOR from the aerobic bacterium *H. thermophilus* (Yoon et al., 1997) (Figure 4). The presence of these enzymes in these aerobic microorganisms remains enigmatic. In aerobic cyanobacteria, PFOR may support metabolism under anoxic conditions (Schmitz et al., 2001). In *Anabaena*, at least one PFOR, produced even in the presence of O_2_, is located in heterocysts, likely O_2_‐depleted compartments (Razquin et al., 1995; Schmitz et al., 2001).

The PFOR from *D. africanus* has a reversible thiol‐disulfide bond in domain VII. Under oxidative conditions, the disulfide bridge forms, protecting the enzyme from O_2_. Under reducing conditions, the disulfide bond is broken and a conformational change occurs, rendering the PFOR much more active (Chabrière et al., 1999; Vita et al., 2008). The PFOR from *Nv*H, which can also form a disulfide bond in the presence of O_2_, was proposed to play a significant role in the dormancy state of this sulfate‐reducing bacterium, characterized by a growth cessation in the presence of O_2_. As an essential enzyme in *Nv*H metabolism, PFOR plays a critical role in this process by quickly transitioning from an inactive to an active form (Ramel et al., 2015).

In *E. coli*, pyruvate oxidation is primarily mediated by either the PFL or the PDH complex. PFOR (YdbK) was unexpectedly found to play a protective role against oxidative stress in this bacterium. By reducing Fld, PFOR and the Fld:NADPH reductase Fpr were proposed to maintain the cellular redox state and restore a reducing environment by reducing various reductases (Nakayama et al., 2013). Recent studies have shown that PFOR activity is maintained under aerobic conditions in vivo. They have also identified PFOR's role in generating NADPH in engineered *E. coli*, through a new pathway that couples PFOR (reducing Fld) to Fpr working in reverse (reducing NADP^+^). This pathway is proposed to be triggered by oxidative stress when NADPH generation from glucose‐6‐phosphate dehydrogenase is insufficient to meet cellular needs during stationary phase under certain conditions (Li, Ye, et al., 2021). This response is induced by modulating NADPH levels, even in the absence of oxidative stress and O_2_ (Krapp et al., 2011).

PFOR's role in oxygenated conditions has been described in *Synechocystis* sp. PCC 6803. This species' photomixotrophic growth in aerobiosis is enabled by PFOR, which remains stable and active in vivo under these conditions, as well as when purified in the presence of O_2_ (Wang et al., 2022). PFOR is essential in these highly reducing conditions because it replaces the PDH complex which is inactivated by a high NADH/NAD^+^ ratio. Under these conditions, cells switch from utilizing NAD(H)‐dependent enzymes to Fd‐dependent enzymes (Wang et al., 2022).

## 7 FUNCTIONAL TRAITS AND METABOLIC INTEGRATION OF PFOR IN EUKARYOTES

Among eukaryotes, PFOR genes have been uncovered in a phylogenetically and physiologically diverse subset of protists (i.e., unicellular eukaryotes). First described in anaerobic eukaryotes, which harbor mitochondrion‐related organelles (MROs) with reduced functions (Hug et al., 2010; Lindmark & Müller, 1973), PFORs were later identified in aerobic plastid‐containing cells (microalgae) (Atteia et al., 2013) and free‐living amoeba (Leger et al., 2013). Despite the growing number of PFOR‐carrying eukaryotes identified (Corre et al., 2026; Stairs et al., 2015), studies on eukaryote PFORs and their metabolic integration remain limited.

### 7.1 PFOR in anaerobic eukaryotes

In anaerobic eukaryotes, also called amitochondriates, PFOR functions as pyruvate decarboxylase (Müller et al., 2012). In the human sexually transmitted parasite *Trichomonas vaginalis*, pyruvate generated by glycolysis is imported into small size H_2_‐producing organelles (hydrogenosomes), and converted by a PFOR into acetyl‐CoA and CO_2_ (Tachezy et al., 2022). The CoA moiety of produced acetyl‐CoA is transferred to succinate by acetate: succinate CoA transferase (ASCT), yielding acetate as an end‐product and succinyl‐CoA (Van Grinsven et al., 2008). A [FeFe]‐hydrogenase reoxidizes Fds, leading to the production of H_2_ that is released as a waste product (Bui & Johnson, 1996). In the human intestinal pathogen *Entamoeba histolytica*, all enzymes involved in energy metabolism are localized in the cytosol. Acetyl‐CoA, generated by PFOR, is converted into acetate and ethanol depending upon the redox state of the cell, via an ADP‐forming acetyl‐CoA synthetase and an aldehyde–alcohol dehydrogenase, respectively (Lo & Reeves, 1978; Reeves et al., 1977). The PFOR in the giardiasis causative agent *Giardia duodenalis* was found to reduce specifically FdI, one of the three natives Fds tested (Townson et al., 1996). PFOR‐produced acetyl‐CoA is converted to acetate and ATP by an ADP‐forming acetyl‐CoA synthetase (Sánchez et al., 2000). The enzyme was found to localize to the cytosol but not to the reduced mitochondrial organelle (Emelyanov & Goldberg, 2011).

### 7.2 PFOR in the free‐living amoeba *A. castellanii*

The amoeba *A. castellanii* is an opportunistic pathogen, able to live under aerobic conditions. When exposed to anoxia, the amoeba quickly undergoes encystation (Turner et al., 2006). Compared with the aerobic form of the amoeba (trophozoites), the cysts exhibit higher PFOR mRNA levels and PFOR activity (Carvalho‐Kelly et al., 2025). Mass spectrometry sequencing of *A. castellanii* mitochondrial proteins identified peptides for PFOR, [FeFe]‐hydrogenase and an acetate: succinate CoA transferase (Leger et al., 2013). PFOR is believed to be pivotal for the amoebozoan metabolism, providing energy for the cyst formation and viability (Carvalho‐Kelly et al., 2022, 2025).

### 7.3 PFOR in the photosynthetic alga *Chlamydomonas reinhardtii*

As yet, the PFOR of the Chlorophyte *C. reinhardtii* is the only algal enzyme to have been biochemically characterized. In vitro studies indicate that low redox potential electrons generated by *C. reinhardtii* PFOR can be transferred to two Fds, FDX1 (PETF) and FDX2 (Van Lis et al., 2013). Production of H_2_ was measured in reconstituted assays comprising recombinant PFOR, a Fd (PETF, FDX2) and the [FeFe] hydrogenase HydA1 (Noth et al., 2013). *C. reinhardtii* is equipped with an extended repertoire of fermentative enzymes, thus providing great metabolic flexibility under anaerobic conditions (Atteia et al., 2006; Hemschemeier & Happe, 2005). For anaerobic pyruvate oxidation, the green alga also has a PFL (Atteia et al., 2006). PFOR, expressed under dark anoxic conditions, was found to localize to the chloroplast, and PFL, a constitutively expressed protein, is present in both energy‐transducing organelles, the chloroplast and the mitochondria (Van Lis et al., 2013).

## 8 CONCLUSIONS

PFORs occupy a central position in anaerobic and microaerophilic metabolism. Over the past few decades, literature has progressively revealed them to be both conserved and remarkably diverse enzymes. Although biochemical, spectroscopic, and structural studies have established a common global structure and TPP‐dependent mechanism, they display extensive variation in subunit organization and domain architecture, which is consistent with their adaptation to different cellular and metabolic contexts over evolutionary time. The range of substrates and redox partners used by these enzymes reveals a continuum from highly pyruvate‐specific PFORs to broadly acting OFORs, as well as a flexible use of ferredoxins, flavodoxins, and rubredoxins as electron donors or acceptors in vitro. This functional plasticity, combined with the underlying structural and evolutionary diversity, highlights the fact that PFORs do not constitute a single canonical enzyme, but rather a more versatile family of TPP‐dependent oxidoreductases that have been remodeled to support a wide variety of metabolic strategies across the tree of life.

The reversible operation of PFOR and its role as a pyruvate synthase have received comparatively little mechanistic attention, and only a few studies have examined the reverse CO_2_ fixing reaction in detail. Recent work mapping the “redox landscape” of several PFORs suggests that despite the architectural diversity of PFORs, the redox potentials of Fe–S clusters were surprisingly conserved in enzymes that work as pyruvate synthase or pyruvate decarboxylase in vivo (Bonitatibus et al., 2025). This raises new questions about how subtle changes in the cofactor environment and partner proteins tune directionality, reversibility, and pathway integration in different organisms. This highlights the reversibility of individual enzymes switching between pyruvate oxidation and CO_2_ fixation in vivo, as a key open question for future studies on the physiological roles, engineering potential, and therapeutic targeting of this enzyme family.

## AUTHOR CONTRIBUTIONS

**Colette Maignet‐Magard:** Investigation; writing – original draft; writing – review and editing. **Myriam Brugna:** Investigation; conceptualization; writing – review and editing. **Ariane Atteia:** Investigation; writing – original draft; writing – review and editing. **Arlette Kpebe:** Writing – review and editing. **Marianne Guiral:** Conceptualization; investigation; writing – original draft; writing – review and editing. **Carole Baffert:** Investigation; conceptualization; writing – original draft; writing – review and editing.

## CONFLICT OF INTEREST STATEMENT

The authors declare no conflicts of interest.

## Supporting information


**Table S1:** Additional information on enzymes exhibiting PFOR activity from Table 1, including specific activity, *K*_m_, redox partners and non‐substrates.
**Figure S1:** Phylogenetic tree of enzymes exhibiting PFOR activity, with bootstrap values.

## Data Availability

The data that support the findings of this study are available from the corresponding author upon reasonable request.
