# Supplementary material for: Exploring the diversity of pyruvate:ferredoxin oxidoreductases
Source: Protein Sci. 2026 Nov 14;35(12):e70853. doi: 10.1002/pro.70853 (PMC13768988; doi:10.1002/pro.70853)
Supplement: Supplementary file 1 [file PRO-35-e70853-s001.docx]

| Organism  (Locus tag) | Specific activity in µmol of pyruvate/min/mg (conditions) | Km pyruvate (µM) | Km CoA (µM) | Protein redox partners | Chemical redox partners | 2-oxoacid **not** substrates | References |
| --- | --- | --- | --- | --- | --- | --- | --- |
| *Aeropyrum pernix*  K1  (APE_2126) | 10.1 (80°C, MV) | 240 | _ | _ | MV | 2-oxoisovalerate | (Nishizawa et al., 2005) |
| *Aeropyrum pernix*  K1  (APE_1473) | 8 (80°C, MV) | 380 | _ | _ | MV | 2-oxoisovalerate | (Nishizawa et al., 2005) |
| *Halobacterium salinarum* (formerly *Halobacterium halobium*) NRC-1  (VNG_1128G) | 4.7 (25°C, pH 8, Cyt reduction assay with Fd) | 100 | _ | Fd | _ | _ | (Kerscher and Oesterhelt, 1982, 1981) |
| *Haloferax volcanii* H26  (HVO_1305) | 1 (42°C, pH 7, MV) | 4790 | 60 | _ | MV | _ | (Kuprat et al., 2021) |
| *Saccharolobus solfataricus* (formerly *Sulfolobus solfataricus*)  (SSO2815) | 7.5 (80°C, pH 8, BV) | 500 | 10.7 | _ | BV | _ | (Alharbi et al., 2022) |
| *Sulfolobus acidocaldarius*  DSM 639  (Saci_2306) | 7 (80°, pH 8, BV)/  10 (70°, pH 9, MV) | 300/210 | 21.5 | Fd | BV, MV | _ | (Alharbi et al., 2022; Witt et al., 2019) |
| *Sulfurisphaera tokodaii* (formerly *Sulfolobus tokodaii*) Strain 7  *OFOR1 –* (STK_23000) | 7.5 (80°C, pH 8.5, MV)/ 30 (80°C, pH 7.5, MV)/ 39 (pH 6.8, Cyt reduction assay with Fd) | 320/280/250 | 74 | Fd | MV | glyoxylate | (Fukuda and Wakagi, 2002; Iwasaki et al., 1995; Yan et al., 2016; Zhang et al., 1996) |
| *Sulfurisphaera tokodaii* (formerly *Sulfolobus tokodaii*) Strain 7  *OFOR2 –* (STK_24350) | 7 (80°C, pH 8.5, MV) | 1600 | _ | _ | MV | _ | (Yan et al., 2016) |
| *Archaeoglobus fulgidus*  DSM 4304  (AF_1701) | 74 (65°C, pH 7.5, MV) | 300 | 20 | Fd | MV, FMN, FAD | 2-oxoglutarate, indol-3-pyruvate, phenylpyruvate, glyoxylate, 3-hydroxypyruvate | (Kunow et al., 1995) |
| *Ignicoccus hospitalis*  KIN4/I  (Igni_1258) | 0.058 (70°C, pH 7.8, MV)* | _ | _ | _ | MV | _ | (Xiao et al., 2021) |
| *Ignicoccus islandicus*  DSM 13165  (EYM_04020) | 0.045 (70°C, pH 7.8, MV)* | _ | _ | Fd | MV | _ | (Xiao et al., 2021) |
| *Methanococcus maripaludis*  JJ/DSM 2067  (MMJJ_13330) | 4.5 (37°C, pH 8.6, MV) | 115 | 5.8 | Rd, Fd (5%) | MV, FMN, FAD | 2-oxoglutarate,  2-oxoisovalerate,  2-oxoisocaproate, phenylpyruvate | (Lin and Whitman, 2004; Lin et al., 2003) |
| *Methanopyrus kandleri*  AV19  (MK0082) | 0.110 (70°C, pH 7.8, MV)* | _ | _ | Fd | MV | _ | (Xiao et al., 2021) |
| *Methanosarcina acetivorans* C2A  (MA_0032) | 19.3 (30°C, pH 7.5, MV)*/ 33.2 (pH 7.2, BV) | 9.3/21 | 4.5 | Fd | BV, MV | _ | (Bonitatibus et al., 2025; Cossu et al., 2024) |
| *Methanosarcina barkeri*  Fusaro DSM 804  (Mbar_A1000) | 20 (37°C, pH 7, Fd)/ 22 (37°C, pH 7, BV) | 70 | 6 | Fd | BV, riboflavin, FMN, FAD | 2-oxoglutarate, phenylpyruvate,  3-hydroxypyruvate, indol-3-pyruvate, glyoxylate,  2-oxaloacetate | (Bock et al., 1997, 1996) |
| *Methanothermobacter marburgensis*  Str. Marburg (formerly *Methanobacterium thermoautotrophicum*)  (MTBMA_c03140) | 18 (65°C, pH 8, BV) | 500 | 30 | _ | BV | 2-oxoglutarate,  2-oxoisovalerate, indol-3-pyruvate | (Tersteegen et al., 1997) |
| *Pyrococcus furiosus*  DSM 3638  (PF0966) | 20/ 22 (80°C, pH 8.4, MV) | 460/1100 | 110 | Fd | MV | 2-oxoglutarate, phenylpyruvate, indol-3-pyruvate | (Blamey and Adams, 1993; Eram et al., 2014; Ma et al., 1997; Menon et al., 1998) |
| *Pyrodictium delaneyi*  Su06  (Pyrde_0268) | 0.140 (70°C, pH 7.8, MV)* | _ | _ | _ | MV | _ | (Xiao et al., 2021) |
| *Pyrolobus fumarii*  DSM 11204  (Pyrfu_0709) | 0.105 (70°C, pH 7.8, MV)* | _ | _ | _ | MV | _ | (Xiao et al., 2021) |
| *Thermococcus guaymasensis*  DSM 11113  ( - ) | 20.2 (80°C, pH 8.4, MV) | 530 | 70 | _ | MV | _ | (Eram et al., 2014) |
| *Thermococcus kodakarensis* KOD1  (TK1983) | 22 (85°C, pH 8.5, MV) | 490 | 115 | Fd | MV | _ | (Nisa et al., 2020) |
| *Thermoproteus tenax*  DSM 2078  (TTX_0209) | 0.043 (70°C, pH 7.8, MV)* | _ | _ | _ | MV | _ | (Xiao et al., 2021) |
| *Anabaena cylindrica*  PCC 7122  (Anacy_0789) | 0.005 (pH 7.9, MV; extract of heterocysts) | 2800 | 30 | Fd | MV | _ | (Bothe et al., 1974; Neuer and Bothe, 1982) |
| *Nostoc* sp. PCC 7120 (formerly *Anabaena* sp. PCC 7120)  ( - ) | 7.8 (pH 7.6, MV) | _ | _ | Fd, Fld (62%) | MV | _ | (Razquin et al., 1995) |
| *Campylobacter jejuni*  NCTC 11168  *(*Cj1476c) | 0.115 (25°C, pH 7.4, BV; cell extract)*/ 2.3 (37°C, pH 8, MV; cell-free extract)*/ 0.57 (23°C, pH 7, BV)* | 300 | _ | Fld | BV, MV | _ | (Hoffman et al., 2007; Kendall et al., 2014; St. Maurice et al., 2007) |
| *Citrobacter sp.*  S77  *(*CIT_000011) | 26.1 (30°C, pH 7, MV) | 120 | 28 | _ | MV | _ | (Takenaka et al., 2017) |
| *Chlorobaculum tepidum* TLS  *(*CT1628) | 0.45 (25°C, pH 7, MV)/ 1 (30°C, pH 7.5, MV)* | 600 | _ | Pyruvate decarboxylation : Rd, Fd (<3%). Pyruvate synthase: Fd, Rd (6%) | MV | _ | (Bonitatibus et al., 2025, 2024; Yoon et al., 2001, 1999) |
| *Clostridium acetobutylicum*  DSM 1731  ( - ) | 25 (25°C, pH 6.9, MB) | 322 | 3.7 | Fd | MB, BV, MV, PMS/MTT, TTC, FAD, FMN | 2-oxaloacetate,  2-oxoglutarate | (Meinecke et al., 1989) |
| *Clostridium sporogenes*  NCIB 10696  ( - ) | 1.3 (pH 8, MV; soluble extract) | _ | _ | _ | BV, MV | _ | (Payne et al., 1990) |
| *Gottschalkia acidurici* (formerly *Clostridium acidurici*)  ( - ) | 1.5 (37°C, pH 7, FAD) | 30/1000 | 25-100 | Fd, Rd (42%) | MV, FMN, FAD, BV, TTC | _ | (Kerscher and Oesterhelt, 1982; Raeburn and Rabinowitz, 1971a, 1971b; Uyeda and Rabinowitz, 1971a, 1971b) |
| *Desulfocurvibacter africanus* (formerly *Desulfovibrio africanus*)  DSM 2603  (Desaf_2186) | 70 (30°C, pH 8.5, MV)/72 (40°C, pH 9, MV) | 2500 | 0.5 | Fd, Fld (13%), Rd (20%) | MV | 2-oxoisovalerate,  2-oxoglutarate | (Chabrière et al., 1999; Pieulle et al., 1999, 1995; Witt et al., 2019) |
| *Desulfomicrobium sp.*  Rod-9  ( - ) | 1.2 (35°C, pH 8.5, MV; cell-free extract) | 2550 | 0.42 | _ | MV | _ | (Kushkevych, 2015) |
| *Desulfovibrio piger*  Vib-7  ( - ) | 0.5 (35°C, pH 8.5, MV; cell-free extract) | 2720 | 0.54 | _ | MV | _ | (Kushkevych, 2015) |
| *Nitratidesulfovibrio vulgaris* (formerly *Desulfovibrio vulgaris*)  Hildenborough  ATCC 29579  (DVU_3025) | _ | _ | _ | _ | _ | _ | (Garczarek et al., 2007) |
| *Nitratidesulfovibrio vulgaris* (formerly *Desulfovibrio vulgaris*)  Miyazaki F IAM 12604  ( - ) | 0.073 (37°C, pH 7, NTB; partially purified)** | 2900 | 32 | Fd, Fld (15%) | NBT, MV, FAD, FMN | 2-oxoglutarate | (Ogata and Yagi, 1986) |
| *Escherichia coli*  K12 BW25113  (BW25113_1378) | 0.018 (30°C, pH 8, BV; partially purified) | 1600 | 16 | Fld, Fd (83%) | MV, BV | _ | (Blaschkowski et al., 1982; Nakayama et al., 2013) |
| *Heliomicrobium modesticaldum* Ice1  (HM1_0807) | _ | _ | _ | Fd | _ | _ | (Walters et al., 2024) |
| *Klebsiella pneumoniae*  M5a1  ( - ) | 6 (30°C, pH 7.5, MV) | 2000 | 4 | Fld, Fd (27%) | MV, BV, phenosafranin, Janus green B | 2-oxoglutarate | (Wahl and Orme-Johnson, 1987) |
| *Neomoorella thermoacetica* (formerly *Moorella thermoacetica* or *Clostridium thermoaceticum*)  ATCC 39073  (Moth_0064) | 15.8 (pH 7.5, MV)* | 300/2000 | 4 | Fd, Rd (67%)(only for pyruvate oxidation reaction) | MV | _ | (Chen et al., 2018; Furdui and Ragsdale, 2000; Menon and Ragsdale, 1997; Wahl and Orme-Johnson, 1987) |
| *Rhodobacter capsulatus* SB 1003  (RCAP_rcc01728) | 15.2 (pH 7.4, MV) | _ | _ | Fld, Fd (75-50%) (2 Fd tested) | MV | _ | (Yakunin and Hallenbeck, 1998) |
| *Rhodospirillum rubrum*  ATCC 11170  (Rru_A2398) | 7.5 (30°C, pH 7.5, MV) | 179 | 9 | Fd | MV | 2-oxoglutarate,  2-oxaloacetate | (Brostedt and Nordlund, 1991; Edgren and Nordlund, 2006; Leroy et al., 2015) |
| *Synechocystis sp.*  PCC 6803  (sll0741) | _ | _ | _ | Fd | MV | _ | (Wang et al., 2022) |
| *Thermoanaerobacter kivui* DSM 2030  (TKV_c04340) | 27.2 (65°C, pH 7.5, Fd)/ 1.8 (65°C, pH 7.5, MB) | 110 | 25.4 | Fd | MB | _ | (Katsyv et al., 2021) |
| *Thermoanaerobacterium saccharolyticum* JW/SL-YS485  (Tsac_0046) | 5 (55°C, pH 7.5, MV; cell extract)* | _ | _ | _ | MV | _ | (Zhou et al., 2015) |
| *Magnetococcus marinus* MC-1  *(*Mmc1_1741) | 8 (30°C, pH 7.5, MV)* | 665 | _ | Fd | MV | _ | (Bonitatibus et al., 2025) |
| *Acetivibrio thermocellus* (formerly *Clostridium thermocellum*)  DSM 1313  (Clo1313_0020–0023 and Clo1313_0673 ) | 4 (55°C, pH 7.5, BV; cell extract)* | _ | _ | _ | BV | _ | (Cui et al., 2019; Xiong et al., 2016) |
| *Helicobacter pylori*  26695  (HP_1110) | 0.127 (25°C, pH 7.4, BV; cell extract)*/ 0.028-0.180 (25°C, pH 7.3, BV; cell extract)*/ 1.2 (30°C, pH 8, MV) | 222/490 | 1.97 | Fld, Fd (27%) | MV, BV, NTZ | 2-oxoglutarate | (Hoffman et al., 2007, 1996; Hughes et al., 1998, 1995; St. Maurice et al., 2007) |
| *Pseudothermotoga hypogea* (formerly *Thermotoga hypogea*)  DSM 11164  (AJ81_03020) | 119 (80°C, pH 8.4, MV) | 130 | 21 | _ | MV | _ | (Eram et al., 2015) |
| *Thermotoga maritima*  DSM 3109  (TM_0017) | 78 (80°C, pH 8.4, MV)/ 87.4 (80°C, pH 8.4, MV)/ 280 (80°C, pH 8.4, Fd) | 400/14500 | 63/340 | Fd | MV | 2-oxoglutarate, phenylpyruvate, indol-3-pyruvate | (Blamey and Adams, 1994; Eram et al., 2015; Kletzin and Adams, 1996) |
| *Aquifex aeolicus* VF5  (aq_1195) | 0.16 (80°C, pH 8, MV; partially purified) | _ | _ | Fd | MV | 2-oxoglutarate | (Prioretti et al., 2023) |
| *Hydrogenobacter thermophilus* TK-6  (HTH_1553) | 1.7 (70°C, pH 7.6, MV)/ 2.5 (70°C, pH 8, MV) | 3450 | 54 | Fd | MV, FMN, FAD | 2-oxoglutarate, 2-oxomalonate,  2-oxoisocaproate, phospho-enolpyruvate, indol-3-pyruvate, phenylpyruvate | (Ikeda et al., 2010, 2006; Yoon et al., 1997) |
| *Chlamydomonas reinhardtii* CC124  (CHLRE_02g095137v5) | 12 (25°C, pH 7, BV)/ 0.45 (pH 8, MV) | 160/1700 | 5.2/700 | Fd | BV, MV, MB, FMN | 2-oxoglutarate | (Noth et al., 2013; Van Lis et al., 2013) |
| *Acanthamoeba castellanii* ATCC 30011  (ACA1_210530) | 7 10^-4^ (pH 7.5, MV; on cysts) | 300 | _ | _ | MV | _ | (Carvalho‐Kelly et al., 2025) |
| *Entamoeba histolytica*  HM-1:IMSS  (EHI_051060) | 0.75 (30°C, pH 7.4, FMN; cell extracts)/ 1.3 (37°C, pH 7, MV)* | 3500 (pH 6)/1500 (pH 7) | 13 (pH 6)/6 (pH 7) | Fd | NBT, MV, TTC, FMN, FAD | 2-oxoglutarate | (Pineda et al., 2010; Reeves et al., 1977) |
| *Giardia duodenalis* (formerly *Giardia intestinalis*)  BRIS/83/HEPU/106  ( - ) | 40.5 (25°C, pH 7.5, MV) | _ | _ | Fd | MV, FMN | 2-oxoglutarate, phenylpyruvate | (Townson et al., 1996) |
| *Trichomonas vaginalis*  Bushby/ CNCD 188  ( - ) | 18.3 (30°C, pH 7, MV)* | 140 | 2.5 | Fd | MV, BV, NBT | _ | (Meza-Cervantez et al., 2011; Steinbüchel and Müller, 1986; Williams et al., 1987) |
| *Tritrichomonas fœtus*  ATCC 30003/ ATCC 30924  ( - ) | 2.13 (30°C, pH 7.1, MV; disrupted hydrogenosome) | _ | _ | Fd | MV | _ | (Docampo et al., 1987; Steinbüchel and Müller, 1986) |

*Table S1: Enzymes exhibiting PFOR activity.*

*Specific activity: for pyruvate decarboxylation activity. The conditions in which the specific activity was determined are indicated in brackets. When the original report indicated the specific activity as µmol of MV or BV/min/mg of protein, the value was halved to obtain the value of activity in µmol of pyruvate/min/mg. * indicates that there was no indication on the definition of the Unit (U).**Assuming that protein solution at 1mg/mL has an absorbance of 1 at 280nm.*

*Protein redox partners: proteins used as acceptor with the percentage of activity relative to that obtained with the best acceptor shown in brackets.*

*Fd: Ferredoxin, Fld: Flavodoxin, Rd: Rubredoxin, Cyt: Cytochrome c, MV: Methylviologen, BV: Benzylviologen, FMN: Flavin mononucleotide, FAD: Flavin adenine dinucleotide, MB: Methylene blue, PMS: Phenazine methosulfate, MTT: 3-(4,5-dimethylthiazol-2-yl)-2,5-diphenyl tetrazolium bromide, TTC: Triphenyl tetrazolium chloride, NBT: Nitro Blue tetrazolium, NTZ: Nitazoxanide*

*Figure S1: Phylogenetic tree of PFORs listed in Table 1, with bootstrap values determined with 1000 replications. The tree was made with MEGA12 software* (Kumar et al., 2024)*. For the multi-subunit PFORs, the gene sequences of all subunits were fused (in the same order as in figure 3A) to establish a more accurate phylogeny, except for the ε subunit of the five-subunit PFORs which was omitted. Accession numbers of the 𝛼-subunits sequences are indicated in Table 1. The symbols indicate domains of life (triangle for Archaea, dot for Bacteria and square for Eukarya)*

Alharbi, F., Knura, T., Siebers, B., Ma, K., 2022. Thermostable and O_2_-Insensitive Pyruvate Decarboxylases from Thermoacidophilic Archaea Catalyzing the Production of Acetaldehyde. Biology 11, 1247. https://doi.org/10.3390/biology11081247

Blamey, J.M., Adams, M.W.W., 1994. Characterization of an Ancestral Type of Pyruvate Ferredoxin Oxidoreductase from the Hyperthermophilic Bacterium, *Thermotoga maritima*. Biochemistry 33, 1000–1007. https://doi.org/10.1021/bi00170a019

Blamey, J.M., Adams, M.W.W., 1993. Purification and characterization of pyruvate ferredoxin oxidoreductase from the hyperthermophilic archaeon *Pyrococcus furiosus*. Biochim. Biophys. Acta, Protein Struct. Mol. Enzymol. 1161, 19–27. https://doi.org/10.1016/0167-4838(93)90190-3

Blaschkowski, H.P., Knappe, J., Ludwig‐Festl, M., Neuer, G., 1982. Routes of Flavodoxin and Ferredoxin Reduction in *Escherichia coli*: CoA‐Acylating Pyruvate: Flavodoxin and NADPH: Flavodoxin Oxidoreductases Participating in the Activation of Pyruvate Formate‐Lyase. Eur. J. Biochem. 123, 563–569. https://doi.org/10.1111/j.1432-1033.1982.tb06569.x

Bock, A., Kunow, J., Glasemacher, J., Schönheit, P., 1996. Catalytic Properties, Molecular Composition and Sequence Alignments of Pyruvate: Ferredoxin Oxidoreductase from the Methanogenic Archaeon *Methanosarcina Barkeri* (Strain Fusaro). Eur. J. Biochem. 237, 35–44. https://doi.org/10.1111/j.1432-1033.1996.0035n.x

Bock, A., Schönheit, P., Teixeira, M., 1997. The iron‐sulfur centers of the pyruvate:ferredoxin oxidoreductase from *Methanosarcina barkeri* (Fusaro). FEBS Lett. 414, 209–212. https://doi.org/10.1016/S0014-5793(97)00998-8

Bonitatibus, S.C., Pham, R.V., Weitz, A.C., Lopéz-Muñoz, M.M., Li, B., Metcalf, W.W., Nair, S.K., Elliott, S.J., 2025. The redox landscape of pyruvate:ferredoxin oxidoreductases reveals often conserved Fe–S cluster potentials. J. Biol. Chem. 301, 110380. https://doi.org/10.1016/j.jbc.2025.110380

Bonitatibus, S.C., Walker, M., Elliott, S.J., 2024. Assessing the role of redox carriers in the reduction of CO_2_ by the oxo-acid: ferredoxin oxidoreductase superfamily. Methods Enzymol. 708, 275–295. https://doi.org/10.1016/bs.mie.2024.10.022

Bothe, H., Falkenberg, B., Nolteernsting, U., 1974. Properties and function of the pyruvate: Ferredoxin oxidoreductase from the blue-green alga *Anabaena cylindrica*. Arch. Microbiol. 96, 291–304. https://doi.org/10.1007/BF00590185

Brostedt, E., Nordlund, S., 1991. Purification and partial characterization of a pyruvate oxidoreductase from the photosynthetic bacterium *Rhodospirillum rubrum* grown under nitrogen-fixing conditions. Biochem. J. 279, 155–158. https://doi.org/10.1042/bj2790155

Carvalho‐Kelly, L.F., Da Costa, R.O., Meyer‐Fernandes, J.R., 2025. Identification of a Pyruvate Ferredoxin Oxidoreductase in *Acanthamoeba castellanii* Cysts: A Key Enzyme in Cyst Energy Homeostasis. Cell Biol. Int. 49, 1319–1328. https://doi.org/10.1002/cbin.70057

Chabrière, E., Charon, M., Volbeda, A., Pieulle, L., Hatchikian, E.C., Fontecilla–Camps, J., 1999. Crystal structures of the key anaerobic enzyme pyruvate:ferredoxin oxidoreductase, free and in complex with pyruvate. Nat. Struct Biol. 6, 182–190. https://doi.org/10.1038/5870

Chen, P.Y.-T., Aman, H., Can, M., Ragsdale, S.W., Drennan, C.L., 2018. Binding site for coenzyme A revealed in the structure of pyruvate:ferredoxin oxidoreductase from *Moorella thermoacetica*. Proc. Natl. Acad. Sci. U.S.A. 115, 3846–3851. https://doi.org/10.1073/pnas.1722329115

Cossu, M., Catlin, D., Elliott, S.J., Metcalf, W.W., Nair, S.K., 2024. Structural organization of pyruvate: ferredoxin oxidoreductase from the methanogenic archaeon *Methanosarcina acetivorans*. Structure 32, 1963-1972.e3. https://doi.org/10.1016/j.str.2024.08.011

Cui, J., Olson, D.G., Lynd, L.R., 2019. Characterization of the *Clostridium thermocellum* AdhE, NfnAB, ferredoxin and Pfor proteins for their ability to support high titer ethanol production in *Thermoanaerobacterium saccharolyticum*. Metab. Eng. 51, 32–42. https://doi.org/10.1016/j.ymben.2018.09.006

Docampo, R., Moreno, S.N., Mason, R.P., 1987. Free radical intermediates in the reaction of pyruvate:ferredoxin oxidoreductase in *Tritrichomonas foetus* hydrogenosomes. J. Biol. Chem. 262, 12417–12420. https://doi.org/10.1016/S0021-9258(18)45219-2

Edgren, T., Nordlund, S., 2006. Two pathways of electron transport to nitrogenase in *Rhodospirillum rubrum* : the major pathway is dependent on the *fix* gene products. FEMS Microbiol. Lett. 260, 30–35. https://doi.org/10.1111/j.1574-6968.2006.00297.x

Eram, M.S., Oduaran, E., Ma, K., 2014. The Bifunctional Pyruvate Decarboxylase/Pyruvate Ferredoxin Oxidoreductase from *Thermococcus guaymasensis*. Archaea 2014, 1–13. https://doi.org/10.1155/2014/349379

Eram, M.S., Wong, A., Oduaran, E., Ma, K., 2015. Molecular and biochemical characterization of bifunctional pyruvate decarboxylases and pyruvate ferredoxin oxidoreductases from *Thermotoga maritima* and *Thermotoga hypogea*. J. Biochem. 158, 459–466. https://doi.org/10.1093/jb/mvv058

Fukuda, E., Wakagi, T., 2002. Substrate recognition by 2-oxoacid:ferredoxin oxidoreductase from *Sulfolobus* sp. strain 7. Biochim. Biophys. Acta, Protein Struct. Mol. Enzymol. 1597, 74–80. https://doi.org/10.1016/S0167-4838(02)00280-7

Furdui, C., Ragsdale, S.W., 2000. The Role of Pyruvate Ferredoxin Oxidoreductase in Pyruvate Synthesis during Autotrophic Growth by the Wood-Ljungdahl Pathway. J. Biol. Chem. 275, 28494–28499. https://doi.org/10.1074/jbc.M003291200

Garczarek, F., Dong, M., Typke, D., Witkowska, H., Hazen, T., Nogales, E., Biggin, M., Glaeser, R., 2007. Octomeric pyruvate-ferredoxin oxidoreductase from *Desulfovibrio vulgaris*. J. Struct. Biol. 159, 9–18. https://doi.org/10.1016/j.jsb.2007.01.020

Hoffman, P.S., Goodwin, A., Johnsen, J., Magee, K., Veldhuyzen Van Zanten, S.J., 1996. Metabolic activities of metronidazole-sensitive and -resistant strains of Helicobacter pylori: repression of pyruvate oxidoreductase and expression of isocitrate lyase activity correlate with resistance. J Bacteriol 178, 4822–4829. https://doi.org/10.1128/jb.178.16.4822-4829.1996

Hoffman, P.S., Sisson, G., Croxen, M.A., Welch, K., Harman, W.D., Cremades, N., Morash, M.G., 2007. Antiparasitic Drug Nitazoxanide Inhibits the Pyruvate Oxidoreductases of *Helicobacter pylori* , Selected Anaerobic Bacteria and Parasites, and *Campylobacter jejuni*. Antimicrob. Agents Chemother. 51, 868–876. https://doi.org/10.1128/AAC.01159-06

Hughes, N.J., Chalk, P.A., Clayton, C.L., Kelly, D.J., 1995. Identification of carboxylation enzymes and characterization of a novel four-subunit pyruvate:flavodoxin oxidoreductase from *Helicobacter pylori*. J. Bacteriol. 177, 3953–3959. https://doi.org/10.1128/jb.177.14.3953-3959.1995

Hughes, N.J., Clayton, C.L., Chalk, P.A., Kelly, D.J., 1998. *Helicobacter pylori porCDAB* and *oorDABC* Genes Encode Distinct Pyruvate:Flavodoxin and 2-Oxoglutarate:Acceptor Oxidoreductases Which Mediate Electron Transport to NADP. J. Bacteriol. 180, 1119–1128. https://doi.org/10.1128/JB.180.5.1119-1128.1998

Ikeda, T., Ochiai, T., Morita, S., Nishiyama, A., Yamada, E., Arai, H., Ishii, M., Igarashi, Y., 2006. Anabolic five subunit-type pyruvate:ferredoxin oxidoreductase from *Hydrogenobacter thermophilus* TK-6. Biochem. Biophys. Res. Commun. 340, 76–82. https://doi.org/10.1016/j.bbrc.2005.11.155

Ikeda, T., Yamamoto, M., Arai, H., Ohmori, D., Ishii, M., Igarashi, Y., 2010. Enzymatic and electron paramagnetic resonance studies of anabolic pyruvate synthesis by pyruvate: ferredoxin oxidoreductase from *Hydrogenobacter thermophilus*. FEBS J. 277, 501–510. https://doi.org/10.1111/j.1742-4658.2009.07506.x

Iwasaki, T., Wakagi, T., Oshima, T., 1995. Ferredoxin-dependent Redox System of a Thermoacidophilic Archaeon, *Sulfolobus* sp. Strain 7. J. Biol. Chem. 270, 17878–17883. https://doi.org/10.1074/jbc.270.30.17878

Katsyv, A., Schoelmerich, M.C., Basen, M., Müller, V., 2021. The pyruvate:ferredoxin oxidoreductase of the thermophilic acetogen, *Thermoanaerobacter kivui*. FEBS Open Bio 11, 1332–1342. https://doi.org/10.1002/2211-5463.13136

Kendall, J.J., Barrero‐Tobon, A.M., Hendrixson, D.R., Kelly, D.J., 2014. Hemerythrins in the microaerophilic bacterium *Campylobacter jejuni* help protect key iron–sulphur cluster enzymes from oxidative damage. Environ. Microbiol. 16, 1105–1121. https://doi.org/10.1111/1462-2920.12341

Kerscher, L., Oesterhelt, D., 1982. Pyruvate : ferredoxin oxidoreductase — new findings on an ancient enzyme. Trends Biochem. Sci. 7, 371–374. https://doi.org/10.1016/0968-0004(82)90118-9

Kerscher, L., Oesterhelt, D., 1981. Purification and Properties of Two 2‐Oxoacid:Ferredoxin Oxidoreductases from *Halobacterium halobium*. Eur. J. Biochem. 116, 587–594. https://doi.org/10.1111/j.1432-1033.1981.tb05376.x

Kletzin, A., Adams, M.W., 1996. Molecular and phylogenetic characterization of pyruvate and 2-ketoisovalerate ferredoxin oxidoreductases from *Pyrococcus furiosus* and pyruvate ferredoxin oxidoreductase from *Thermotoga maritima*. J. Bacteriol. 178, 248–257. https://doi.org/10.1128/jb.178.1.248-257.1996

Kumar, S., Stecher, G., Suleski, M., Sanderford, M., Sharma, S., Tamura, K., 2024. MEGA12: Molecular Evolutionary Genetic Analysis Version 12 for Adaptive and Green Computing. Mol. Biol. Evol. 41, msae263. https://doi.org/10.1093/molbev/msae263

Kunow, J., Linder, D., Thauer, R.K., 1995. Pyruvate: ferredoxin oxidoreductase from the sulfate-reducing *Archaeoglobus fulgidus*: molecular composition, catalytic properties, and sequence alignments. Arch. Microbiol. 163, 21–28. https://doi.org/10.1007/BF00262199

Kuprat, T., Ortjohann, M., Johnsen, U., Schönheit, P., 2021. Glucose Metabolism and Acetate Switch in Archaea: the Enzymes in *Haloferax volcanii*. J. Bacteriol. 203. https://doi.org/10.1128/JB.00690-20

Kushkevych, I.V., 2015. Kinetic properties of pyruvate ferredoxin oxidoreductase of intestinal sulfate-reducing bacteria *Desulfovibrio piger* Vib-7 and *Desulfomicrobium* sp. Rod-9. Pol. J. Microbiol. 64, 107–114. https://doi.org/10.33073/pjm-2015-016

Leroy, B., De Meur, Q., Moulin, C., Wegria, G., Wattiez, R., 2015. New insight into the photoheterotrophic growth of the isocytrate lyase-lacking purple bacterium *Rhodospirillum rubrum* on acetate. Microbiology 161, 1061–1072. https://doi.org/10.1099/mic.0.000067

Lin, W., Whitman, W.B., 2004. The importance of porE and porF in the anabolic pyruvate oxidoreductase of *Methanococcus maripaludis*. Arch. Microbiol. 181, 68–73. https://doi.org/10.1007/s00203-003-0629-1

Lin, W.C., Yang, Y.-L., Whitman, W.B., 2003. The anabolic pyruvate oxidoreductase from *Methanococcus maripaludis*. Arch. Microbiol. 179, 444–456. https://doi.org/10.1007/s00203-003-0554-3

Ma, K., Hutchins, A., Sung, S.-J.S., Adams, M.W.W., 1997. Pyruvate ferredoxin oxidoreductase from the hyperthermophilic archaeon, *Pyrococcus furiosus* , functions as a CoA-dependent pyruvate decarboxylase. Proc. Natl. Acad. Sci. U.S.A. 94, 9608–9613. https://doi.org/10.1073/pnas.94.18.9608

Meinecke, B., Bertram, J., Gottschalk, G., 1989. Purification and characterization of the pyruvate-ferredoxin oxidoreductase from *Clostridium acetobutylicum*. Arch. Microbiol. 152, 244–250. https://doi.org/10.1007/BF00409658

Menon, A.L., Hendrix, H., Hutchins, A., Verhagen, M.F.J.M., Adams, M.W.W., 1998. The δ-Subunit of Pyruvate Ferredoxin Oxidoreductase from *Pyrococcus* *furiosus* Is a Redox-Active, Iron−Sulfur Protein: Evidence for an Ancestral Relationship with 8Fe-Type Ferredoxins. Biochemistry 37, 12838–12846. https://doi.org/10.1021/bi980979p

Menon, S., Ragsdale, S.W., 1997. Mechanism of the *Clostridium thermoaceticum* Pyruvate:Ferredoxin Oxidoreductase: Evidence for the Common Catalytic Intermediacy of the Hydroxyethylthiamine Pyropyrosphate Radical. Biochemistry 36, 8484–8494. https://doi.org/10.1021/bi970403k

Meza-Cervantez, P., González-Robles, A., Cárdenas-Guerra, R.E., Ortega-López, J., Saavedra, E., Pineda, E., Arroyo, R., 2011. Pyruvate : ferredoxin oxidoreductase (PFO) is a surface-associated cell-binding protein in *Trichomonas vaginalis* and is involved in trichomonal adherence to host cells. Microbiology 157, 3469–3482. https://doi.org/10.1099/mic.0.053033-0

Nakayama, T., Yonekura, S.-I., Yonei, S., Zhang-Akiyama, Q.-M., 2013. *Escherichia coli* pyruvate:flavodoxin oxidoreductase, YdbK - regulation of expression and biological roles in protection against oxidative stress. Genes Genet. Syst. 88, 175–188. https://doi.org/10.1266/ggs.88.175

Neuer, G., Bothe, H., 1982. The pyruvate: Ferredoxin oxidoreductase in heterocysts of the cyanobacteriuim *Anabaena cylindrica*. Biochim. Biophys. Acta 716, 358–365. https://doi.org/10.1016/0304-4165(82)90028-9

Nisa, K., Ashraf, S., Siddiqui, M.A., Taj, N., Rehman, H.-U., Bano, A., Rashid, N., 2020. Purification and Characterization of a Thermostable Pyruvate Ferredoxin Oxidoreductase/Pyruvate Decarboxylase from *Thermococcus kodakaraensis*. Pak. J. Zool. 52. https://doi.org/10.17582/journal.pjz/20191018081056

Nishizawa, Y., Yabuki, T., Fukuda, E., Wakagi, T., 2005. Gene expression and characterization of two 2‐oxoacid:ferredoxin oxidoreductases from *Aeropyrum pernix* K1. FEBS Lett. 579, 2319–2322. https://doi.org/10.1016/j.febslet.2004.11.117

Noth, J., Krawietz, D., Hemschemeier, A., Happe, T., 2013. Pyruvate:Ferredoxin Oxidoreductase Is Coupled to Light-independent Hydrogen Production in *Chlamydomonas reinhardtii*. J. Biol. Chem. 288, 4368–4377. https://doi.org/10.1074/jbc.M112.429985

Ogata, M., Yagi, T., 1986. Pyruvate Dehydrogenase and the Path of Lactate Degradation in *Desulfovibrio vulgaris* Miyazaki F1. J. Biochem. 100, 311–318. https://doi.org/10.1093/oxfordjournals.jbchem.a121717

Payne, M.J., Woods, L.F.J., Gibbs, P., Cammack, R., 1990. Electron paramagnetic resonance spectroscopic investigation of the inhibition of the phosphoroclastic system of *Clostridium sporogenes* by nitrite. J. Gen. Microbiol. 136, 2067–2076. https://doi.org/10.1099/00221287-136-10-2067

Pieulle, L., Charon, M., Bianco, P., Bonicel, J., Pétillot, Y., Hatchikian, E.C., 1999. Structural and kinetic studies of the pyruvate–ferredoxin oxidoreductase/ferredoxin complex from *Desulfovibrio africanus*. Eur. J. Biochem. 264, 500–508. https://doi.org/10.1046/j.1432-1327.1999.00648.x

Pieulle, L., Guigliarelli, B., Asso, M., Dole, F., Bernadac, A., Hatchikian, E.C., 1995. Isolation and characterization of the pyruvate-ferredoxin oxidoreductase from the sulfate-reducing bacterium *Desulfovibrio africanus*. Biochim. Biophys. Acta, Protein Struct. Mol. Enzymol. 1250, 49–59. https://doi.org/10.1016/0167-4838(95)00029-T

Pineda, E., Encalada, R., Rodríguez‐Zavala, J.S., Olivos‐García, A., Moreno‐Sánchez, R., Saavedra, E., 2010. Pyruvate:ferredoxin oxidoreductase and bifunctional aldehyde–alcohol dehydrogenase are essential for energy metabolism under oxidative stress in *Entamoeba histolytica*. FEBS J. 277, 3382–3395. https://doi.org/10.1111/j.1742-4658.2010.07743.x

Prioretti, L., D’Ermo, G., Infossi, P., Kpebe, A., Lebrun, R., Bauzan, M., Lojou, E., Guigliarelli, B., Giudici-Orticoni, M.-T., Guiral, M., 2023. Carbon Fixation in the Chemolithoautotrophic Bacterium *Aquifex aeolicus* Involves Two Low-Potential Ferredoxins as Partners of the PFOR and OGOR Enzymes. Life 13, 627. https://doi.org/10.3390/life13030627

Raeburn, S., Rabinowitz, J.C., 1971a. Pyruvate: Ferredoxin oxidoreductase: I. The pyruvate-CO_2_ exchange reaction. Arch. Biochem. Biophys. 146, 9–20. https://doi.org/10.1016/S0003-9861(71)80036-X

Raeburn, S., Rabinowitz, J.C., 1971b. Pyruvate: Ferredoxin oxidoreductase: II. Characteristics of the forward and reverse reactions and properties of the enzyme. Arch. Biochem. Biophys. 146, 21–33. https://doi.org/10.1016/S0003-9861(71)80037-1

Razquin, P., Schmitz, S., Peleato, M.L., Fillat, M.F., Gomez-Moreno, C., Böhme, H., 1995. Differential activities of heterocyst ferredoxin, vegetative cell ferredoxin, and flavodoxin as electron carriers in nitrogen fixation and photosynthesis in *Anabaena* sp. Photosynth. Res. 43, 35–40. https://doi.org/10.1007/BF00029460

Reeves, R.E., Warren, L.G., Susskind, B., Lo, H.S., 1977. An energy-conserving pyruvate-to-acetate pathway in *Entamoeba histolytica*. Pyruvate synthase and a new acetate thiokinase. J. Biol. Chem. 252, 726–731. https://doi.org/10.1016/S0021-9258(17)32778-3

St. Maurice, M., Cremades, N., Croxen, M.A., Sisson, G., Sancho, J., Hoffman, P.S., 2007. Flavodoxin:Quinone Reductase (FqrB): a Redox Partner of Pyruvate:Ferredoxin Oxidoreductase That Reversibly Couples Pyruvate Oxidation to NADPH Production in *Helicobacter pylori* and *Campylobacter jejuni*. J. Bacteriol. 189, 4764–4773. https://doi.org/10.1128/JB.00287-07

Steinbüchel, A., Müller, M., 1986. Anaerobic pyruvate metabolism of *Tritrichomonas foetus* and *Trichomonas vaginalis* hydrogenosomes. Mol. Biochem. Parasitol. 20, 57–65. https://doi.org/10.1016/0166-6851(86)90142-8

Takenaka, M., Yoon, K.-S., Matsumoto, T., Ogo, S., 2017. Acetyl-CoA production by encapsulated pyruvate ferredoxin oxidoreductase in alginate hydrogels. Bioresour. Technol. 227, 279–285. https://doi.org/10.1016/j.biortech.2016.12.051

Tersteegen, A., Linder, D., Thauer, R.K., Hedderich, R., 1997. Structures and Functions of Four Anabolic 2‐Oxoacid Oxidoreductases in *Methanobacterium Thermoautotrophicum*. Eur. J. Biochem. 244, 862–868. https://doi.org/10.1111/j.1432-1033.1997.00862.x

Townson, S.M., Upcroft, J.A., Upcroft, P., 1996. Characterisation and purification of pyruvate:ferredoxin oxidoreductase from *Giardia duodenalis*. Mol. Biochem. Parasitol. 79, 183–193. https://doi.org/10.1016/0166-6851(96)02661-8

Uyeda, K., Rabinowitz, J.C., 1971a. Pyruvate-Ferredoxin Oxidoreductase III. Purification and properties of the enzyme. J. Biol. Chem. 246, 3111–3119. https://doi.org/10.1016/S0021-9258(18)62202-1

Uyeda, K., Rabinowitz, J.C., 1971b. Pyruvate-Ferredoxin Oxidoreductase IV. Studies on the reaction mechanism. J. Biol. Chem. 246, 3120–3125. https://doi.org/10.1016/S0021-9258(18)62203-3

Van Lis, R., Baffert, C., Couté, Y., Nitschke, W., Atteia, A., 2013. *Chlamydomonas reinhardtii* Chloroplasts Contain a Homodimeric Pyruvate:Ferredoxin Oxidoreductase That Functions with FDX1. Plant Physiol. 161, 57–71. https://doi.org/10.1104/pp.112.208181

Wahl, R.C., Orme-Johnson, W.H., 1987. Clostridial pyruvate oxidoreductase and the pyruvate-oxidizing enzyme specific to nitrogen fixation in *Klebsiella pneumoniae* are similar enzymes. J. Biol. Chem. 262, 10489–10496. https://doi.org/10.1016/S0021-9258(18)60987-1

Walters, K.A., Redding, K.E., Golbeck, J.H., 2024. Identification and characterization of the low molecular mass ferredoxins involved in central metabolism in *Heliomicrobium modesticaldum*. Photosynth. Res. 162, 251–271. https://doi.org/10.1007/s11120-023-01069-z

Wang, Y., Chen, X., Spengler, K., Terberger, K., Boehm, M., Appel, J., Barske, T., Timm, S., Battchikova, N., Hagemann, M., Gutekunst, K., 2022. Pyruvate:ferredoxin oxidoreductase and low abundant ferredoxins support aerobic photomixotrophic growth in cyanobacteria. eLife 11, e71339. https://doi.org/10.7554/eLife.71339

Williams, K., Lowe, P.N., Leadlay, P.F., 1987. Purification and characterization of pyruvate: ferredoxin oxidoreductase from the anaerobic protozoon *Trichomonas vaginalis*. Biochem. J. 246, 529–536. https://doi.org/10.1042/bj2460529

Witt, A., Pozzi, R., Diesch, S., Hädicke, O., Grammel, H., 2019. New light on ancient enzymes – *in vitro* CO_2_ Fixation by Pyruvate Synthase of *Desulfovibrio africanus* and *Sulfolobus acidocaldarius*. FEBS J. 286, 4494–4508. https://doi.org/10.1111/febs.14981

Xiao, L., Liu, G., Gong, F., Cai, Z., Li, Y., 2021. The reductive carboxylation activity of heterotetrameric pyruvate synthases from hyperthermophilic archaea. Biochem. Biophys. Res. Commun. 572, 151–156. https://doi.org/10.1016/j.bbrc.2021.07.091

Xiong, W., Lin, P.P., Magnusson, L., Warner, L., Liao, J.C., Maness, P.-C., Chou, K.J., 2016. CO_2_ -fixing one-carbon metabolism in a cellulose-degrading bacterium *Clostridium thermocellum*. Proc. Natl. Acad. Sci. U.S.A. 113, 13180–13185. https://doi.org/10.1073/pnas.1605482113

Yakunin, A.F., Hallenbeck, P.C., 1998. Purification and characterization of pyruvate oxidoreductase from the photosynthetic bacterium *Rhodobacter capsulatus*. Biochim. Biophys. Acta, Bioenerg. 1409, 39–49. https://doi.org/10.1016/S0005-2728(98)00145-5

Yan, Z., Maruyama, A., Arakawa, T., Fushinobu, S., Wakagi, T., 2016. Crystal structures of archaeal 2-oxoacid:ferredoxin oxidoreductases from *Sulfolobus tokodaii*. Sci. Rep. 6, 33061. https://doi.org/10.1038/srep33061

Yoon, K.-S., Bobst, C., Hemann, C.F., Hille, R., Tabita, F.R., 2001. Spectroscopic and Functional Properties of Novel 2[4Fe-4S] Cluster-containing Ferredoxins from the Green Sulfur Bacterium *Chlorobium tepidum*. J. Biol. Chem. 276, 44027–44036. https://doi.org/10.1074/jbc.M107852200

Yoon, K.-S., Hille, R., Hemann, C., Tabita, F.R., 1999. Rubredoxin from the Green Sulfur Bacterium *Chlorobium tepidum* Functions as an Electron Acceptor for Pyruvate Ferredoxin Oxidoreductase. J. Biol. Chem. 274, 29772–29778. https://doi.org/10.1074/jbc.274.42.29772

Yoon, K.-S., Ishii, M., Kodama, T., Igarashi, Y., 1997. Purification and characterization of pyruvate:ferredoxin oxidoreductase from *Hydrogenobacter thermophilus* TK-6. Arch. Microbiol. 167, 275–279. https://doi.org/10.1007/s002030050443

Zhang, Q., Iwasaki, T., Wakagi, T., Oshima, T., 1996. 2-Oxoacid:Ferredoxin Oxidoreductase from the Thermoacidophilic Archaeon, *Sulfolobus* sp. Strain 7. J. Biochem. 120, 587–599. https://doi.org/10.1093/oxfordjournals.jbchem.a021454

Zhou, J., Olson, D.G., Lanahan, A.A., Tian, L., Murphy, S.J.-L., Lo, J., Lynd, L.R., 2015. Physiological roles of pyruvate ferredoxin oxidoreductase and pyruvate formate-lyase in *Thermoanaerobacterium saccharolyticum* JW/SL-YS485. Biotechn. Biofuels 8, 138. https://doi.org/10.1186/s13068-015-0304-1
